# A target trial emulation of the CONFIRM study with an extension to subgroups: an example for relapsing-remitting multiple sclerosis

**DOI:** 10.1007/s10654-026-01379-1

**Published:** 2026-03-18

**Authors:** Stefan Verweij, Maarten J. Bijlsma, Katrien Oude Rengerink, Jan Hillert, Lars Forsberg, Elena Flavia Mouresan, Anna Glaser, Robert James Fox, Eelko Hak, Peter Mol

**Affiliations:** 1 Unit of PharmacoTherapy, Epidemiology and Economics, Groningen Research Institute of Pharmacy, Groningen, the Netherlands; 2 Dutch Medicines Evaluation Board, Utrecht, The Netherlands; 3 Department of Clinical Neuroscience, Karolinska Institute, Stockholm, Sweden; 4 Cleveland Clinic, Mellen Center for Multiple Sclerosis Treatment and Research, Neurological Institute, Cleveland, OH USA; 5 Department of Clinical Pharmacy and Pharmacology, University Medical Center Groningen, University of Groningen, Groningen, The Netherlands

**Keywords:** Target trial emulation, Multiple sclerosis, Subgroups, Retrospective cohort study, Real-world evidence, Observational study

## Abstract

Conclusions of target trial emulations (TTEs) are often limited to trial-like populations, leaving knowledge gaps for neglected subpopulations. We propose a two-step TTE approach to create methodologically-justified evidence for these subpopulations. Using the CONFIRM trial as an illustrative example, we first emulated the trial using strict eligibility criteria, and extended the analysis to a pragmatic scenario including trial-ineligible subgroups, allowing comparison of treatment effectiveness between these two study populations. CONFIRM studied efficacy of dimethyl fumarate (DMF) and glatiramer acetate (GA) in reducing relapses in 709 relapsing-remitting multiple sclerosis (RRMS) patients. A retrospective cohort study was designed using the Swedish multiple sclerosis registry. Outcomes were the annualized relapse rate (ARR) and the probability of a relapse, adjusted for covariates and overlap weights. Using strict eligibility criteria, 288 RRMS patients were included, whereas the pragmatic scenario comprised 1831 patients. The ARR ratio for DMF versus GA was 0.78 (95%CI 0.59–1.05) in CONFIRM, 0.75 (95%CI: 0.44–1.29) in the strict scenario, and 0.58 (95% CI: 0.36–0.94) in the pragmatic scenario. Similarly, the hazard ratio was 0.92 (95%CI 0.70–1.22), 1.05 (95%CI: 0.83–1.33) and 0.56 (95%CI: 0.46–0.67), respectively. *Post-hoc* analysis implied existence of calendar bias. Comparable results between CONFIRM and the strict scenario were observed, while the second step demonstrates how methodological advantages of TTEs can be extended to excluded subgroups. This study can be used as guiding example for future observational studies that wish to incorporate the TTE framework, as well as embrace the wider variety in the patient population often ignored in experimental settings.

**Supplementary Information:**

The online version contains supplementary material available at https://doi.org/10.1007/s10654-026-01379-1.

## Introduction

Relapsing-remitting multiple sclerosis (RRMS) is the most common phenotype of multiple sclerosis in adults aged twenty to fifty, with a higher occurrence in women [1–3]. Early-onset (< 18 years of age) or late-onset (> 50 years of age) disease is less common [4, 5]. Prevalence is higher in North America and Europe, although this could be a proxy for access to healthcare in these regions [6, 7]. These patient-characteristics may explain why trial-populations generally consist of adult Caucasians and often exclude geriatric populations or other subpopulations in whom benefits or risks might differ [8]. As not all patient groups have been included in trials that support approval of disease modifying therapies, gaps of knowledge of the causal effect for these patient groups exist [8, 9].

In theory, these knowledge gaps could be filled by obtaining evidence from additional randomized controlled trials or from real-world data. Additional randomized trials are, however, often challenging and costly to conduct, and may not be prioritized in case of no obvious expected effect modification in patients groups excluded from the trials. Comparative effectiveness analysis based on observational data is more feasible and might provide sufficient evidence to exclude major differences, however, it is prone to biases, such as selection bias and immortal time bias [10–12]. For this reason, Hernan and Robins developed the target trial emulation framework to help specify the causal question and to better avoid methodological pitfalls that can be observed in observational studies [13].

By customizing the eligibility criteria of the observational study, one can choose to closely emulate the (hypothetical) target trial by studying a highly similar study population or approach the real-world population by dropping some of the often strict eligibility criteria applied in experimental trials. In this latter, more pragmatic scenario, subgroups are included which are otherwise neglected when applying strict trial criteria. Outcome estimates for these subgroups, i.e., specific subsets of participants in our study population, could then be extrapolated to their resembling subpopulations [14].

Therefore, we present a two-step approach by emulating an existing randomized placebo active controlled trial, CONFIRM [15], following two scenarios. In the first step, we adhere to a strict trial-like scenario aimed to obtain outcome estimates similar to the original target trial, thus increasing confidence in the validity of our study design. In the second step, using the same design, a more pragmatic approach is followed by dropping eligibility criteria, and thus including usually excluded subgroups, to see if, and how these outcome estimates differ from the original target trial and the strict trial-like scenario. If the outcomes from the strict scenario are in line with the target trial, any differences in outcomes observed in the second step are hypothesized to be caused by the different study population, that is, the subgroup(s) ineligible for the original CONFIRM trial.

## Methods

### Data source

A registry-based study was designed using data from the Swedish Multiple Sclerosis registry (SMSreg) to emulate the active controlled part of the CONFIRM randomized trial using the target trial emulation framework (Table 1) [13, 16]. CONFIRM was a phase 3 study to investigate efficacy and safety of twice-daily dimethyl fumarate (BID) as compared to placebo, with a reference comparator, glatiramer acetate (GA). The study was designed and powered for comparison against placebo. In CONFIRM, 709 BID and GA patients with relapsing-remitting multiple sclerosis (RRMS) were enrolled from 28 countries. The primary and secondary endpoints in the trial were the annualized relapse rate estimated over a 2 year period (96 weeks) and the proportion of subjects who had relapsed over the same period. CONFIRM was selected, since it was a relatively large, recent and active controlled randomized trial studying two first-line treatments, a detailed rationale can be found in the Supplementary Information (Appendix 1). SMSreg, active since 2001, is a government funded registry covering 84% the Swedish MS population, and used by all Swedish neurological departments [16]. The registry tracks data of patient characteristics, demographics, clinical visits, treatments and relapses, amongst others.

This study was approved by the Swedish ethical committee (2024-01458-01). The protocol can be accessed at the real-world data catalogue of the European Medicines agency (EUPAS1000000135) [17]. TARGET checklist can be found in the Supplementary Information (Appendix 2) [18].

### Population

Two different populations were considered in this cohort study. In the strict scenario, the eligibility criteria were defined as those published in the study protocol of the CONFIRM trial by Fox et al. and applicable to the registry data, to mimic the estimates from the CONFIRM trial as closely as possible [19]. In the pragmatic scenario, we dropped three key eligibility criteria, i.e., the age criterion, the criterion for the Expanded Disability Status Scale (EDSS) – a scale to describe disease severity – and the criterion for the number of relapses experienced in the year prior to baseline, henceforth referred to as the pre-baseline annualized relapse rate (ARR). These criteria were dropped to include the following subgroups: (1) older patients (> 55 years), (2) patients with severe disability (EDSS > 5), and (3) relapse-free patients (pre-baseline ARR = 0), respectively. We also estimated the effects in six additional combinations of the three eligibility criteria, plus three scenarios where we only studied the specific subgroups as the effect within subgroups might be different. In total, 11 scenarios were considered. Primary and secondary progressive multiple sclerosis (PPMS and SPMS) patients at baseline were excluded.

### Treatment and follow-up

The target trial emulation was conducted in Swedish RRMS patients initiating either dimethyl fumarate (DMF) or GA between first occurrence in the registry (14 May 1998) and 19 June 2024. Treatment initiation was defined as having no prior history with the assigned treatment available in the registry and being on the assigned treatment for at least two days to mimic the intention-to-treat (ITT) analysis, as one day was assumed to be an administration error in the registry. Study start, also known as baseline or time zero, was the date of first prescription. In the observational analog of the intention to treat analysis, patients were followed until death, loss to follow-up, study end (96 weeks) or end of data collection (19 June 2024), whichever occurred first. The per-protocol (PP) analysis was similar to our observational analogue of the ITT analysis in all but one aspect, since, in the PP analysis, discontinuation of treatment was also included as a censoring event. For both the ITT and PP analysis, consistency and positivity were assumed.

### Outcomes

The primary endpoint of the target trial emulation was the ARR over a course of 96 weeks of follow-up, where a relapse was either directly determined by a healthcare professional or self-reported and retroactively confirmed by the physician and added to the registry. The secondary endpoint was the probability of experiencing a first relapse in a 96-week period. Due to the observational nature of the data, the observation of the EDSS variable closest to baseline was used as observation at baseline. A time-window of maximum of one year prior to baseline was applied as it was assumed that EDSS values within this one year window were still relevant at baseline.

**Table 1:** Components of the target trial, the strict trial-like emulation and the pragmatic emulation

Eligibility	CONFIRM RCT	Strict scenario	Pragmatic scenario
	Patients diagnosed with RMSS: • aged 18–55, • EDSS score between 0 and 5, • at least one positive relapse in year prior to study entry with a prior MRI demonstrating a lesion, or at least one MRI demonstrating a Gd-enhancing lesion in the 6 weeks prior to study entry. For detailed exclusion criteria, see the original trial protocol.^*^	Patients diagnosed with RMSS: • aged 18–55, • EDSS score between 0 and 5, • at least one positive relapse in year prior to study entry. For detailed exclusion criteria, see the original study protocol.^†^	Patients diagnosed with RRMS. Exclusion criteria are similar to strict scenario.
Treatment strategy	Patients received either oral placebo, twice daily oral 120 mg DMF, three times daily oral 120 mg DMF or subcutaneous daily injections of 20 mg GA over the course of 96 weeks.	Patients initiated either oral DMF or subcutaneous glatiramer acetate. Dosing regimens not specified, but assumed to be in accordance to Swedish prescription guidelines (i.e., 240 mg DMF and 20 mg GA daily).	Patients initiated either oral DMF or subcutaneous glatiramer acetate. Dosing regimens not specified, but assumed to be in accordance to Swedish prescription guidelines (i.e., 240 mg DMF and 20 mg GA daily).
Treatment assignment	Eligible patients were randomly assigned 1:1:1:1 to twice daily DMF, three times daily DMF, GA and placebo. Randomization took place across all study sites, stratified by site.	Overlap weights based on propensity scores were used to create a pseudo-population where treatment assignment approaches equal likelihood (PS ≈ 0.5); mimicking equal likelihood of treatment assignment during the randomization process. Extreme PS are downweighed.	Overlap weights based on propensity scores were used to create a pseudo-population where treatment assignment approaches equal likelihood (PS ≈ 0.5); mimicking equal likelihood of treatment assignment during the randomization process. Extreme PS are downweighed.
Follow-up	From time of randomization until 96 weeks of follow-up.	ITT: Patients were followed from the first dose (= time zero) until study end (96 weeks), death, loss to follow-up or end of data collection; whichever occurred first. PP: patients were followed from the first dose (= time zero) until study end (96 weeks), death, loss to follow-up, end of data collection or treatment discontinuation; whichever occurred first.	ITT: Patients were followed from the first dose (= time zero) until study end (96 weeks), death, loss to follow-up or end of data collection; whichever occurred first. PP: patients were followed from the first dose (= time zero) until study end (96 weeks), death, loss to follow-up, end of data collection or treatment discontinuation; whichever occurred first.
Outcomes	Relapses defined as new or recurrent neurologic symptoms not associated with fever or infection, lasting at least 24 h, and accompanied by new objective neurological findings upon examination by the examining neurologist.	Relapses as registered by a physician in the Swedish multiple sclerosis registry.	Relapses as registered by a physician in the Swedish multiple sclerosis registry.
Causal contrast	ITT and PP.	Observational analogue of ITT (= treatment initiators) and PP.	Observational analogue of ITT (= treatment initiators) and PP.
Statistical analysis	• Number of relapses over the course of 96 weeks, presented through the annualized relapse rates and rate ratio; estimated using a negative binomial regression model. • The proportion relapsed over the course of 2 years, estimated using a Cox Proportional Hazards model. Both estimators adjusted for bias due to confounding using the covariates, age, EDSS at baseline, and baseline relapse rate.	• Number of relapses over the course of 96 weeks, presented through the annualized relapse rates and rate ratio; estimated using a negative binomial regression model. • The proportion relapsed over the course of 2 years, estimated using a Cox Proportional Hazards model. Both estimators adjusted for bias due to confounding using the covariates, age, EDSS at baseline, and baseline relapse rate; both estimators weighted using overlap weights.	• Number of relapses over the course of 96 weeks, presented through the annualized relapse rates and rate ratio; estimated using a negative binomial regression model. • The proportion relapsed over the course of 2 years, estimated using a Cox Proportional Hazards model. Both estimators adjusted for bias due to confounding using the covariates, age, EDSS at baseline, and baseline relapse rate; both estimators weighted using overlap weights.

*DMF* Dimethyl fumarate,* EDSS* Expanded Disability Status Scale,* GA* Glatiramer acetate,* ITT* Intention to treat,* PP* Per protocol, *RCT* Randomized controlled trial,* RRMS* Relapsing-remitting multiple sclerosis

^*^DOI: https://doi.org/10.1056/NEJMoa1206328

^†^EUPAS1000000135

### Statistical analysis

#### Analysis of the emulated target trial

Descriptive analyses were conducted for all covariates. Continuous variables were summarized using means and standard deviations, while categorical variables were presented as frequencies and percentages. Differences between the two treatment arms were evaluated using t-tests for continuous variables and chi-square tests for categorical variables. Missing values for the EDSS score were assumed to be missing at random (MAR) and imputed using multiple imputation (MICE) prior to applying the time-window and eligibility criteria, with treatment, age, sex, pre-baseline ARR, calendar year, treatment history (since registry enrollment) of both first and second line therapies (Supplementary Information Table S1), time to relapse, the ARR after 96 weeks and the study duration as predictors, and predictive mean matching as method [20]. Since the imputation had to be performed prior to treatment assignment, missingness per strategy, as recommended by TARGET, was not feasible [21]. Time to first relapse was analyzed using a Cox proportional hazards model, including a term for treatment and adjusted for baseline EDSS, age and the number of relapses in the year prior to baseline, as these were the covariates adjusted for in the CONFIRM trial. As opposed to what was stated in our study protocol, we used overlap weights based on propensity scores (PS) instead of inverse probability of treatment weights (IPtW), to estimate the average treatment effect of the overlap population (ATO) instead of the average treatment effect (ATE). The PS is the probability that the patient receives the treatment of interest given their covariates, where we used the same covariates as in our outcome models [22]. The overlap weighting method weighs each patient by the probability of being assigned to the other treatment group  PS*(1-PS), hence penalizing patients with extreme PS and inflating the weight of patients where treatment assignment is equally likely [23]. Equal chance of being assigned to a treatment group is the objective of randomization, and thus the ATO estimate is better suited in comparison to the ATE when the goal is to strictly emulate an RCT. Both adjusted and unadjusted Kaplan-Meier curves were plotted to estimate the probability of experiencing a first relapse over the course of the study. The ARR was analyzed using a negative binomial regression model adjusted for the same covariates and weighted using the same overlap weights. Multicollinearity was examined using generalized variance inflation factors [24].

#### Comparison of the emulations to the target trial

The scenarios were compared to the target trial through the evaluation of clinical relevance and support of three binary metrics as described by Franklin et al. (2019), i.e. (1) “regulatory agreement” or statistical (in)significance agreement, where the estimates and confidence intervals (CIs) of both the RCT and the TTE scenario are on the same side of the null, (2) estimate agreement, where the point estimate of the TTE scenario falls within the CI of the RCT, and (3) standardized difference agreement, where the standardized difference between the two point estimates is less than 1.96 standard errors (p-value > 0.05) [25].

#### Sensitivity and supplementary analyses

To get better insight into the impact of deviating from the assumptions made in our main analysis, we performed multiple sensitivity and supplementary analyses:

1. Change of analysis model: while we used the negative binomial regression model for the annualized relapse rate as to adhere to the analysis plan of the original CONFIRM trial, a Poisson regression was also applied as relapses follow a Poisson distribution [26].
2. Change of definition of conversion rate: we assessed the impact of the uncertainty in the definition of the conversion rate to SPMS and included patients with a registered conversion in the year and a half prior to baseline. Some of these included SPMS patients may actually have progressed after baseline, since the date of conversion is determined retrospectively and hard to link to a specific date [27].
3. We applied the g-formula for a static treatment strategy as opposed to the PS-based method in the main analysis to assess robustness of the outcome estimates, as the g-formula is not dependent on the treatment assignment model used to calculate the overlap weights, because it predicts counterfactual outcomes for each patient under different treatment scenarios [28]. Moreover, using the g-formula, we can compare its average treatment effect (ATE) estimate to the average treatment effect estimate of the overlap population (ATO) obtained in our main analysis.
4. We reduced the time window of the EDSS at baseline variable from one year prior to baseline to half a year prior to baseline, since this would give us a better approximation of the real EDSS value at baseline, although less patients will become eligible.
5. We only selected relapses confirmed by the neurologist, thus excluding self-reported relapses which are more prone to measurement bias.
6. We added additional covariates (sex, history of DMT usage and calendar period) to our PS- and outcome models to assess robustness. Calendar period was defined as the year in which treatment was initiated. Additionally, in a *post-hoc* analysis, only history of DMT usage was added to the set of covariates from the main analysis,

In addition, two *post-hoc* analyses were performed to assess bias due to calendar year. First, a subgroup analysis was done to see if patients initiating GA after 2012 had different outcome estimates compared to patients initiating GA prior to 2012. The GA subgroup that initiated treatment after 2012 was hypothesized to be more comparable to the DMF arm, since DMF was authorised for the European market in 2014 [29]. Moreover, Schoenfeld residuals were plotted against calendar year to assess violation of proportional hazards [30].

## Results

### Patients

**Fig. 1:**
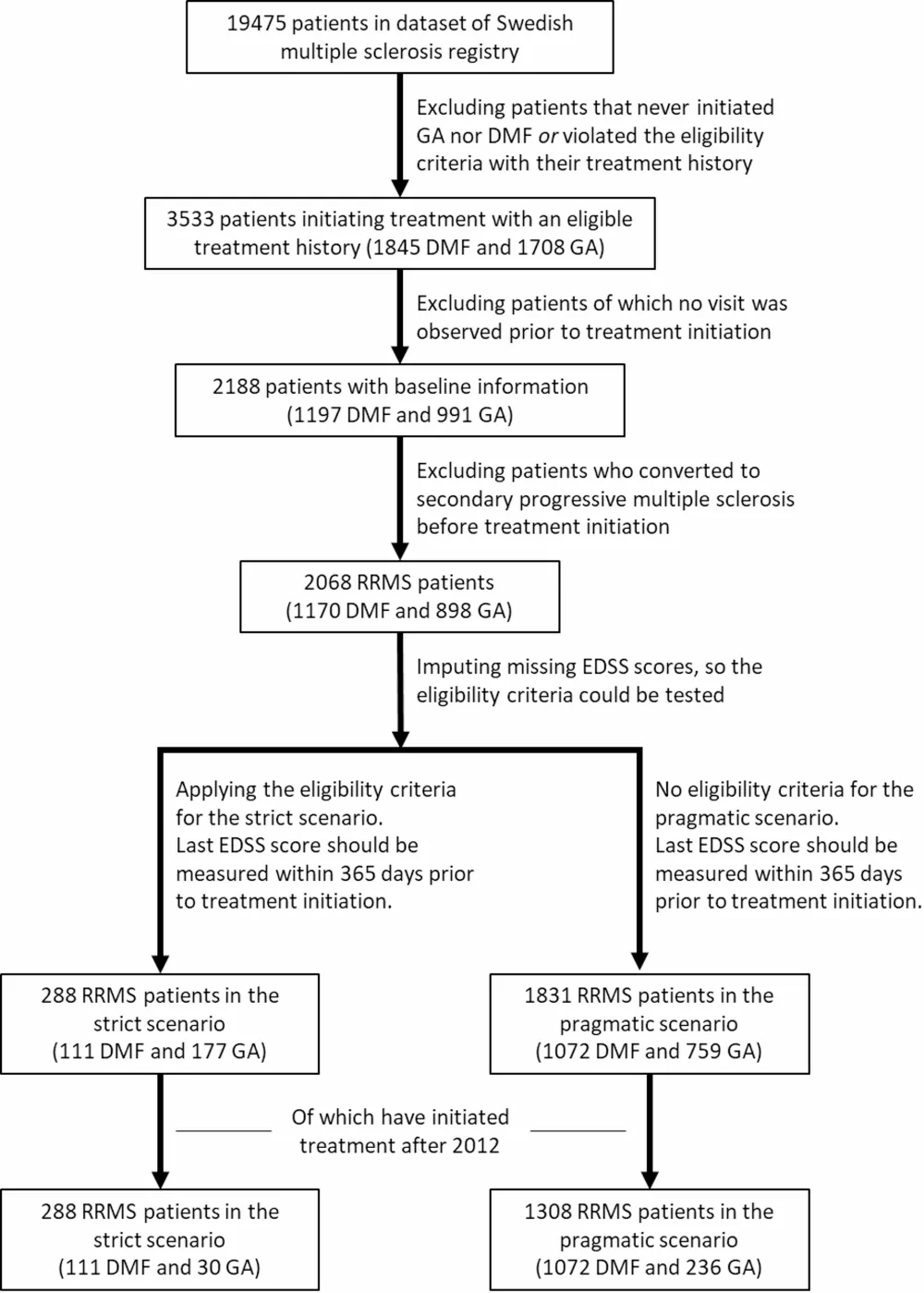
Flowchart of patient selection in the Swedish multiple sclerosis registry for both the strict trial-like emulation and the pragmatic scenario. *DMF* Dimethyl fumarate, *EDSS* Expanded disability status scale, *GA* Glatiramer acetate, *RRMS* Relapsing-remitting multiple sclerosis

Of the 288 eligible patients in the strict scenario, 111 initiated DMF and 177 initiated GA, whereas in CONFIRM 709 patients initiated treatment (359 DMF and 350 GA) (Fig. 1). In the pragmatic scenario, 1831 patients were eligible (1072 DMF and 759 GA). Missingness of the EDSS score was 18.05% prior to imputation and prior to applying the eligibility criteria. Baseline characteristics of the two scenarios, together with the baseline characteristics of the original CONFIRM trial are given in Table 2. Baseline characteristics of the overlap population were roughly similar, although the pre-baseline ARR became more equal between the two arms after application of the overlap weights (Supplementary Information Table S2 and Figure S1). The two treatment arms had little overlap in the period of treatment initiation (Supplementary Information Figure S2). For the other scenarios, six additional patients were included when dropping the criterion for the EDSS score (2 DMF and 4 GA patients), and 12 additional patients when dropping the age criterion (2 DMF and 10 GA patients) (Supplementary Information Table S3). When dropping the pre-baseline ARR ≥ 1 criterion, 1361 patients became eligible (871 DMF and 490 GA patients). Of the three specific subgroup scenarios, only the subgroup with no relapses in the year prior to baseline contained enough patients for analysis. Reasons for censoring were equally distributed (Supplementary Information Table S5). Characteristics of the censored population differed from those that completed the study (Supplementary Information Table S6 and Figure S3).

**Table 2:** Baseline characteristics of the original CONFIRM trial, the strict scenario and the pragmatic scenario

	CONFIRM trial		Strict scenario			Pragmatic scenario		
	DMF	GA	DMF	GA	*p*	DMF	GA	*p*
*n*	359	350	111	177		1072	759	
age (mean (SD))	37.8 (9.4)	36.7 (9.1)	34.4 (8.2)	36.1 (8.3)	0.10	38.2 (10.7)	39.7 (10.8)	**0.002**
Sex (%)*					0.52			**0.002**
Female (%)	245 (68)	247 (71)	81 (73)	137 (77)		754 (70)	585 (77)	
Male (%)	114 (32)	103 (29)	30 (27)	41 (23)		318 (30)	174 (23)	
EDSS at baseline (mean (SD))	2.6 (1.2)	2.6 (1.2)	1.7 (1.1)	1.9 (1.3)	0.38	1.5 (1.4)	1.8 (1.4)	**< 0.001**
Number of relapses in previous 12 months (mean (SD))	1.3 (0.6)	1.4 (0.6)	1.3 (0.5)	1.5 (0.8)	**0.01**	0.1 (0.4)	0.4 (0.8)	**< 0.001**
Number of relapses in previous 12 months (%)*					**0.02**			**< 0.001**
0	*na*	*na*	0 (0)	0 (0)		957 (89)	568 (75)	
1	*na*	*na*	84 (76)	125 (71)		87 (8)	132 (17)	
2	*na*	*na*	24 (22)	30 (17)		24 (2)	36 (5)	
3	*na*	*na*	3 (3)	16 (9)		3 (0)	16 (2)	
4	*na*	*na*	0 (0)	7 (4)		1 (0)	7 (1)	
Number of pre-baseline DMT treatments (%)*					**0.01**			**< 0.001**
0	258 (72)	247 (71)	87 (78)	110 (62)		748 (70)	452 (60)	
1	*na*	*na*	19 (17)	55 (31)		225 (21)	259 (34)	
2	*na*	*na*	4 (4)	13 (7)		77 (7)	46 (6)	
3	*na*	*na*	1 (1)	0 (0)		17 (2)	2 (0)	
4	*na*	*na*	0 (0)	0 (0)		5 (0)	0 (0)	
Of which firstline therapies (%)*					**0.007**			**< 0.001**
0	*na*	*na*	89 (80)	111 (63)		758 (71)	458 (60)	
1	*na*	*na*	18 (16)	56 (32)		253 (24)	268 (35)	
2	*na*	*na*	4 (4)	11 (6)		55 (5)	31 (4)	
3	*na*	*na*	0 (0)	0 (0)		6 (1)	2 (0)	
Of which secondline therapies (%)*					0.45			**0.005**
0	*na*	*na*	108 (97)	174 (98)		1019 (95)	739 (97)	
1	*na*	*na*	2 (2)	3 (2)		37 (3)	19 (3)	
2	*na*	*na*	1 (1)	0 (0)		16 (1)	1 (0)	
Years since diagnosis (SD)	4.9 (5.1)	4.4 (4.7)	1.6 (3.8)	2.4 (3.9)	0.07	2.8 (5.0)	3.5 (5.3)	**0.006**

*na* values indicate that the value was not reported in the CONFIRM trial

*DMF* Dimethyl fumarate,* DMT* Disease modifying therapy,* EDSS* Expanded disability status scale,* GA* Glatiramer acetate,* SD* Standard deviation

*Due to rounding of the pooled estimates after imputation, the percentages may not add up to exactly 100%, and the summation of categorical variables may not be equal to *n*

### Annualized relapse rate

**Fig. 2:**
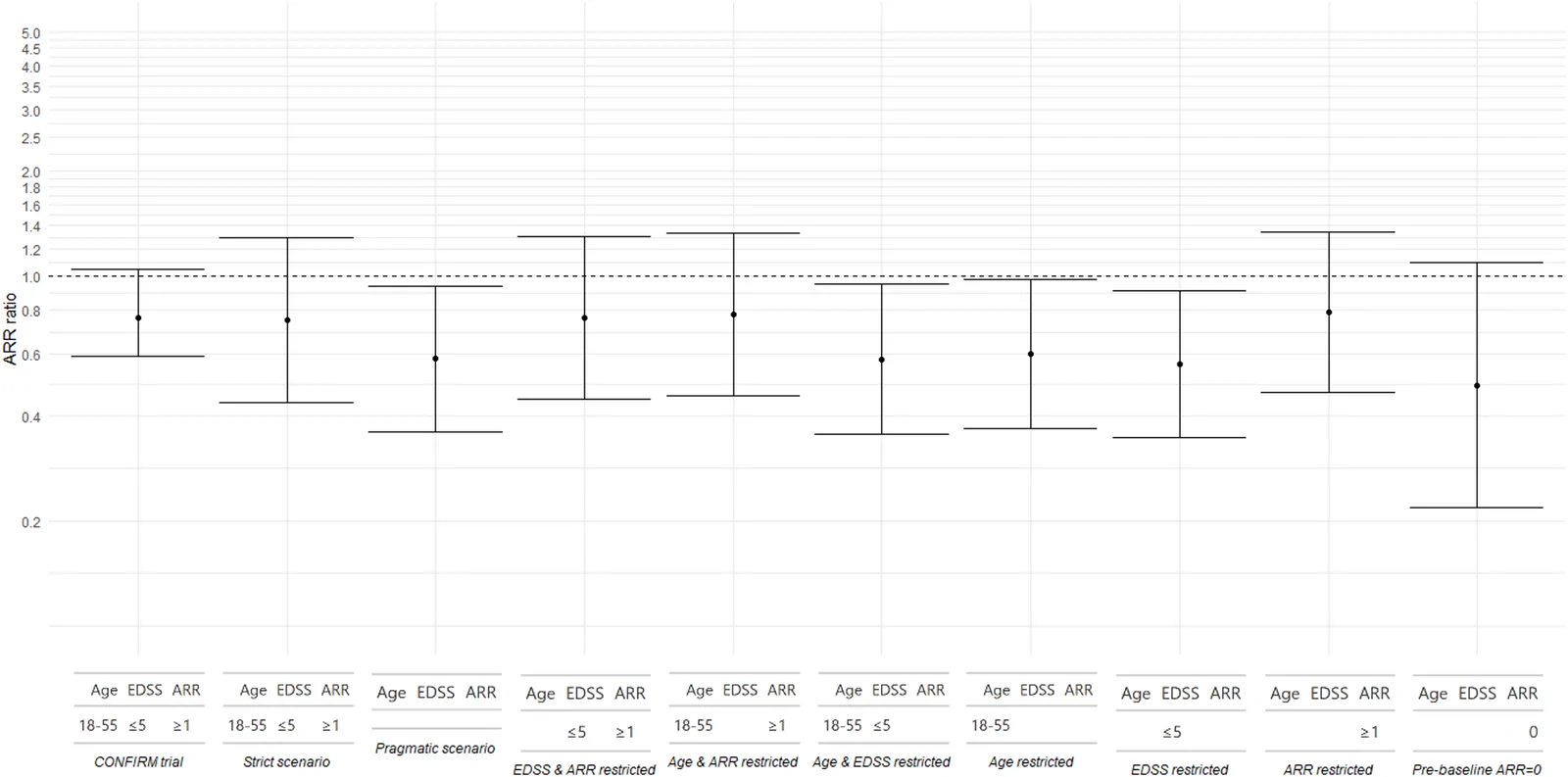
ARR ratios with their 95% confidence intervals of DMF versus GA for the different scenarios, estimated through negative binomial regression models. The dotted line (ratio = 1) indicates the null line, with smaller ratios favoring DMF, and bigger ratios favoring GA. *ARR *Annualized relapse rate, *DMF *Dimethyl fumarate, *EDSS *Expanded Disability Status scale, *GA G*latiramer acetate

In the observational analogue of our ITT analysis, the ARR over 96 weeks was 0.88 (95%CI 0.25–3.09) in the DMF cohort of the strict scenario (*n* = 111), compared to 0.20 (95%CI 0.08–0.50) in the pragmatic scenario (*n* = 1072) (Table 3). Similarly, for the GA cohorts, the ARR over 96 weeks was 1.17 (95%CI 0.32–4.26, *n* = 177) and 0.34 (95%CI 0.13–0.89, *n* = 759) for the two respective scenarios (Table 3). The ratios for DMF versus GA were 0.75 (95%CI: 0.44–1.29) in the strict scenario and 0.58 (95% CI: 0.36–0.94) in the pragmatic scenario. While the absolute rates from the CONFIRM trial seemed to be more in line with the pragmatic scenario, the relative rate ratios were more in line with the strict scenario (Fig. 2). The negative binomial regression models failed to converge for scenarios with a small sample size ($\:n\le\:306$).

### Probability of experiencing a first relapse

**Fig. 3:**
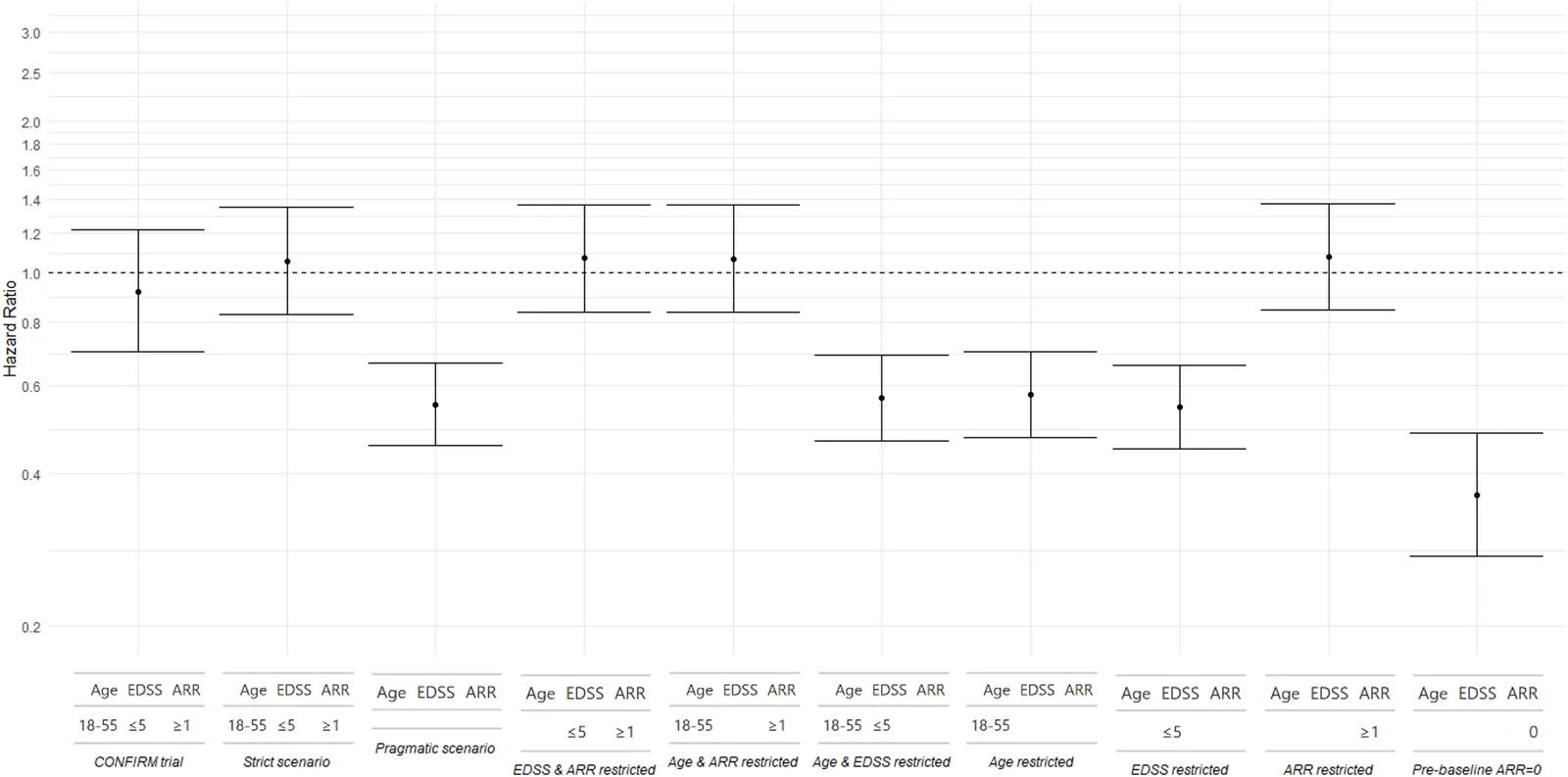
Hazard ratios of time to first relapse with their 95% confidence intervals of DMF versus GA for the different scenarios, estimated through Cox proportional hazards models. The dotted line (ratio = 1) indicates the null line, with smaller ratios favoring DMF, and bigger ratios favoring GA. *ARR *Annualized relapse rate, *DMF *Dimethyl fumarate, *EDSS *Expanded disability status scale, *GA *Glatiramer acetate

**Fig. 4:**
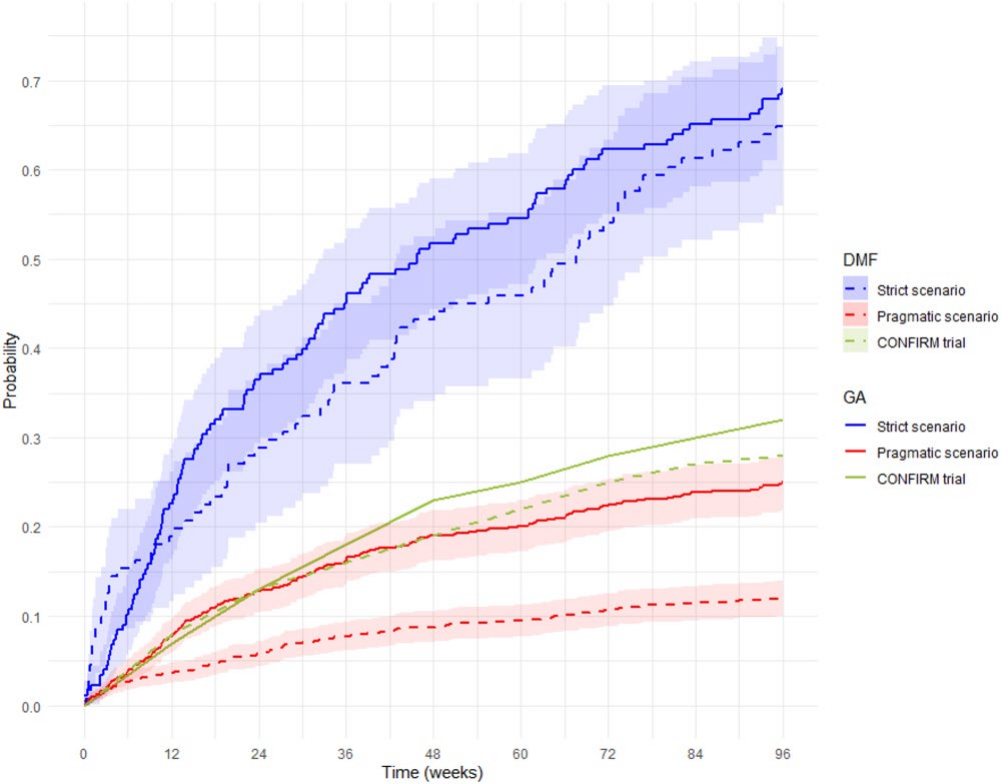
Kaplan-Meier curve showing the probability of experiencing a first relapse since baseline (time zero) for the original CONFIRM trial (green), and the strict (blue) and pragmatic (red) scenarios. The dotted lines represent the DMF cohorts, the solid lines the GA cohorts. *DMF *Dimethyl fumarate, *GA *Glatiramer acetate

In the observational analogue of our ITT analysis, the hazard ratio (HR) of experiencing a first relapse over a course of 96 weeks was 1.05 (95%CI 0.83–1.33) in the strict scenario, compared to 0.92 (95%CI 0.70–1.22) in the CONFIRM trial and 0.56 (95%CI 0.46–0.67) in the pragmatic scenario (Table 3; Fig. 3). Larger effect sizes were observed in the relapse-free subpopulation (pre-baseline ARR = 0) (Fig. 3). The Kaplan-Meier curves indicate that the probability of experiencing a relapse is lower for DMF patients compared to GA patients in the original CONFIRM trial, the strict and the pragmatic scenario (Fig. 4). In all the scenarios where we excluded patients with no relapses in the year prior to baseline, the risk and probability of experiencing a relapse was higher as compared to the CONFIRM trial (Table 3, Supplementary Information Figures S4-S5). Adjusted Kaplan-Meier curves indicate the same, although, and similar to the Cox proportional hazards model, the probability of experiencing a relapse is slightly higher for DMF patients in the strict scenario (Supplementary Information Figures S6-S8).

The binary metrics for both the ARR ratio and the HR indicated that scenarios that only contained patients with recent relapse activity (pre-baseline ARR ≥ 1) were more in line with CONFIRM as compared to the scenarios that included relapse-free patients (pre-baseline ARR = 0) (Table 4). Crude analysis showed similar results for scenarios that contained only patients with recent relapse activity and in the relapse-free subgroup, but results deviated in the scenarios that contained both types of patients (pre-baseline ARR ≥ 0) (Supplementary Information Table S7). The results of the per protocol analysis are given in Supplementary information Table S8.

### Sensitivity and subgroup analysis

Five out of the six sensitivity analyses (changing the statistical model to Poisson regression, including SPMS patients, application of the g-formula, adjusting the EDSS at baseline window, and filtering on physician-confirmed relapses) indicated robustness of our approach (Supplementary Information Tables S9-S13). In the sensitivity analysis with additional covariates, the estimates deviated from the main analysis, although multicollinearity was introduced (Supplementary Information Table S14 and Figures S9 and S10). The effectiveness of DMF was slightly more pronounced when only adding treatment history as additional covariate to the ARR model as compared to the main analysis, and GA was estimated to perform slightly better for the time-to-first-relapse. *Post-hoc* subgroup analysis confirmed existence of calendar bias, as observed in the comparison of the covariates and probability of experiencing a first relapse, but showed robust estimates for the annualized relapse rate ratios (Supplementary Information Figures S12 and S13, and Tables S15 and S16). Schoenfeld residuals indicated violation of proportionality (Supplementary Information Figure S14).

**Table 3:** Outcome estimates of the primary and secondary analysis

ITT	CONFIRM			Strict scenario			Pragmatic scenario			EDSS & ARR restricted			Age & ARR restricted			Age & EDSS restricted			pre-baseline ARRs restricted			Age restricted			EDSS restricted			pre-baseline ARR = 0		
Eligibility criteria^§^	Age	EDSS	ARR	Age	EDSS	ARR	Age	EDSS	ARR	Age	EDSS	ARR	Age	EDSS	ARR	Age	EDSS	ARR	Age	EDSS	ARR	Age	EDSS	ARR	Age	EDSS	ARR	Age	EDSS	ARR
	18–55	≤ 5	≥ 1	18–55	≤ 5	≥ 1					≤ 5	≥ 1	18–55		≥ 1	18–55	≤ 5				≥ 1	18–55				≤ 5				0^†^
n	704			288			1831			300			294			1649			306			1675			1794			1525		
Person-years	1296			530			3371			552			541			3034			563			3084			3303			2808		
ARR GA	0.29 [0.23;0.35]			1.17 (0.32;4.26)			0.34 [0.13;0.89]			1.19 [0.36;3.90]			1.17 [0.32;4.21]			0.28 [0.09;0.85]			1.18 [0.36;3.86]			0.28 [0.09;0.85]			0.33 [0.13;0.87]			0.25 [0.51;1.22]		
ARR DMF	0.22 [0.18;0.28]			0.88 [0.25;3.09]			0.20 [0.08;0.50]			0.91 [0.29;2.85]			0.91 [0.26;3.16]			0.16 [0.06;0.48]			0.93 [0.30;2.92]			0.17 [0.06;0.50]			0.19 [0.07;0.47]			0.12 [0.03;0.55]		
ARRR	0.78 [0.59;1.05]			0.75 [0.44;1.29]			0.58 [0.36;0.94]			0.76 [0.44;1.31]			0.78 [0.46;1.33]			0.58 [0.35;0.95]			0.79 [0.47;1.34]			0.60 [0.37;0.98]			0.56 [0.35;0.91]			0.49 [0.22; 1.10]		
Proportion relapsed within 2 years (GA)	32%			67%			19%			66%			66%			20%			66%			19%			19%			12%		
Proportion relapsed within 2 years (DMF)	29%			69%			12%			68%			69%			13%			68%			13%			12%			5%		
HR	0.92 [0.70;1.22]			1.05 [0.83;1.33]			0.56 [0.46;0.67]			1.06 [0.84;1.33]			1.06 [0.84;1.34]			0.57 [0.48;0.69]			1.06 [0.84;1.34]			0.58 [0.48;0.70]			0.55 [0.46;0.67]			0.37 [0.28;0.50]		

^§^The three eligibility criteria are (1) age at baseline between 18 and 55 year, (2) an EDSS score at baseline between 0 and 5, and (3) having had at least one relapse in the year prior to baseline

^†^This is an adjusted criteria where a patient had no relapses in the year prior to baseline

The 95% confidence interval is depicted between brackets

*ARR A*nnualized relapse rate, *ARRR A*nnualized relapse rate ratio, *EDSS *Expanded disability status scale, *HR *Hazard ratio,* ITT *Intention to treat

**Table 4:** Binary metrics to evaluate agreement between estimates from the respective scenario and those reported by the CONFIRM trial

ITT		Strict scenario			Pragmatic scenario			EDSS & ARR restricted			Age & ARR restricted			Age & EDSS restricted			pre-baseline ARRs restricted			Age restricted			EDSS restricted			pre-baseline ARR = 0		
Eligbility criteria ^§^		Age	EDSS	ARR	Age	EDSS	ARR	Age	EDSS	ARR	Age	EDSS	ARR	Age	EDSS	ARR	Age	EDSS	ARR	Age	EDSS	ARR	Age	EDSS	ARR	Age	EDSS	ARR
		18–55	≤ 5	≥ 1					≤ 5	≥ 1	18–55		≥ 1	18–55	≤ 5				≥ 1	18–55				≤ 5				0^†^
Statistical insignficance agreement	ARRR	✓			X			✓			✓			X			✓			X			X			✓		
	HR	X			X			X			X			X			X			X			X			X		
Estimate agreement	ARRR	✓			X			✓			✓			X			✓			✓			X			X		
	HR	✓			X			✓			✓			X			✓			X			X			X		
Standardized difference agreement	ARRR	✓			✓			✓			✓			✓			✓			✓			✓			✓		
	HR	✓			✓			✓			✓			✓			✓			✓			✓			✓		

Statistical insignificance agreement was defined as having the point estimate and the CI limits on the same side of the null as compared with the CONFIRM trial. Estimate agreement was defined as whether the point estimate of the scenario fell within the 95%CI of the CONFIRM trial. Standardized difference agreement was defined as the difference between the two sample means being less than 1.96 standard errors apart:$\:1.96>\left|z\right|={\left|\right(\theta\:}_{RCT}-{\theta\:}_{scenario})/\sqrt{{\sigma\:}_{RCT}^{2}+{\sigma\:}_{scenario}^{2}}|$

*ARR* Annualized relapse rate,* ARRR* Annualized relapse rate ratio,* EDSS* Expanded disability status Scale,* HR* Hazard ratio

^§^The three eligibility criteria are (1) age at baseline between 18 and 55 year, (2) an EDSS score at baseline between 0 and 5, and (3) having had at least one relapse in the year prior to baseline

^†^This is an adjusted eligibility criteria where a patient had no relapses in the year prior to baseline as opposed to CONFIRM’s criteria of having at least one relapse in the year prior to baseline

## Discussion

We followed a two-step approach in emulating the CONFIRM trial using the Swedish multiple sclerosis registry. We applied strict eligibility criteria in the first step to accurately emulate the target trial and gain confidence in the study design, data and analysis, and followed a more pragmatic approach in the second step to study the effectiveness in the population better reflecting real-world users, which included subpopulations previously excluded from pivotal trials. A similar setup was described by Becher et al. (2023) using the Swedish heart failure registry to describe differences between strict-trial like and pragmatic study populations [31].

In the strict scenario, the ARR ratio estimate in the real world setting in Sweden mimics the ratio observed in the CONFIRM target trial, although the point estimate came with a broader confidence interval due to the smaller sample size as compared to the original trial, which was an international study covering 200 sites in 28 countries. Possible differences in outcome estimates might therefore also be partially explained by the multinational patient population in CONFIRM, as opposed to the Swedish patient population covered in SMSreg, although baseline characteristics between the target trial and trial-like scenario were similar.

The hazard ratio in the strict scenario was in line with original trial results as well, although the point estimate differed, possibly due to effect sizes being close to zero [32]. The HR in the pragmatic scenario notably increased and deviated from those reported in the CONFIRM trial and strict scenario, which seemed to be mainly the result of calendar bias. Subgroup analyses on calendar year were performed to get insight into violation of proportionality. The subgroup where the GA arm initiated treatment after 2012, HR estimates of the pragmatic scenario were more in line with the strict scenario and original trial results, implying violation of proportionality. Analysis on the Schoenfeld residuals confirmed this observation. A potential explanation is a change in time to first-confirmed-relapse around the period 2010–2014, when the 2010 revision of the McDonald criteria was published [33]. ARR ratios were less affected in the subgroup analysis, since rate ratios summarize the overall event rate and are therefore less sensitive to time-specific hazards. Thus, especially given time-to-event outcomes, we recommend concurrency between treatment arms when designing target trial emulations.

Previously published literature applied the three statistical metrics used in this study to evaluate the success of the emulation [25, 34]. However, these metrics alone are not sufficient to determine if an emulation has been successful, as each has their limitations. Both estimate agreement and standardized difference agreement fail to incorporate the uncertainty that comes with the target trial estimate. For these two metrics, a TTE would more easily agree with RCT estimates when substantial uncertainty is involved in the trial (large CIs) as opposed to RCT estimates with narrow CIs, as there is a smaller chance of overlap in the latter. Moreover, statistical significance agreement is disproportionately harsh when estimates (or their CI limits) lay close to the null, as was the case with CONFIRM. A rate ratio with an upper CI limit of 0.99 in the emulation and 1.01 in the RCT would fail statistical significance agreement, although they both indicate similar effectiveness. Therefore, we argue that, in any case, statistical metrics should be accompanied by an a priori defined threshold for clinical relevance of differences, where the latter should take into account the purpose of the emulation and the acceptability of any differences and/or uncertainty. For example, if side effects are comparable, treating physicians might be interested in just superiority or non-inferiority of one treatment versus another, whereas in cost-effectiveness research, the magnitude of the relative effect sizes might play a more substantial role. Hence, we recommend building a framework in which consensus on clinical relevance related to study purpose is quantified to allow scientifically sound interpretation of trial emulation comparability.

In our recent publication, we compared observational studies with randomized controlled trials in multiple sclerosis, and showed that dimethyl fumarate outperformed glatiramer acetate, confirming our observations [35]. However, it is important to acknowledge the inherent differences between observational data and experimental settings. First of all, not all eligibility criteria from the CONFIRM trial were applicable to our strict scenario, such as exclusion of patients with HIV infection [19], since this information was not captured in SMSreg. MRI information was not considered in this study either, neither to confirm internal nor external validity (e.g., as eligibility criterion or exploratory outcome); although active lesions are known to be correlated with relapses (Supplementary Information Appendix 3) [36]. Second, the absolute ARRs observed in the strict scenario differed from the original trial results, while for the pragmatic scenario they were more in line with the trial. This may indicate that relapses are registered differently in observational settings as compared to experimental trials. However, in our observational study, the possible measurement bias would be the same for both cohorts, and hence the relative rate ratios remain unaffected. Third, the observational and experimental settings also differ due to the lack of overlap in calendar year between the two cohorts as stated earlier. After the introduction of oral DMTs, treatment initiation of GA diminished, due to improved convenience and higher satisfaction rates of oral therapies as compared to injectables [37, 38]. As a result, the more historic GA group may have experienced a different standard of care, was exposed to different diagnostic criteria and had less treatment alternatives. This violation of the positivity assumption threatens the validity of the estimates presented in this study, and may hinder clinical interpretation of the comparison between DMF and GA.

In both the strict and pragmatic scenarios, the two cohorts differed in more baseline characteristics, most of which were adjusted for in our analysis. Calendar period, treatment history and sex were additionally adjusted for in a sensitivity analysis. Unfortunately, this resulted in multicollinearity which negatively affected reliable interpretation. Moreover, crude analysis led to similar estimates within the strict trial-like scenario, possibly due to the limited variation in covariates within this homogeneous trial-like population; whereas greater covariate variability in the more heterogeneous population of the pragmatic scenario likely led to stronger influence of confounding adjustment. However, the setting of multiple sclerosis – with relapses as outcome of interest – was deliberately chosen due to the small set of known confounders [39, 40]. We thereby assumed that the effect of a differing bias due to confounding, because of differing underlying confounding structures, between the pragmatic scenario and the strict trial-like scenario is negligible. As a result of the limited variation in covariates within the scenarios with small homogeneous populations, regression models failed to converge. Furthermore, bias due to informative censoring may have been present, but censoring reasons were not available in the study data. Therefore, model estimates should be interpreted with more caution for these scenarios. Although, for the strict trial-like scenario, the estimates are consistent with the original CONFIRM trial.

Misclassification bias may have been introduced by conversion from the RRMS to the SPMS phenotype around baseline, although our SPMS sensitivity analysis showed estimates in line with the main analysis. Furthermore, we reversed the swap of primary and secondary endpoints as stated in the study protocol, because – while the protocol states otherwise – bias in health-seeking behaviour is the same for both the rate ratio as well as the time-to-first-event endpoints. Therefore, adhering to the endpoints as stated in the original CONFIRM trial protocol was preferred. Additionally, the original CONFIRM trial was not designed to test superiority or noninferiority of DMF versus GA. While the ARR ratio and the HR between DMF and GA were reported in the supplementary information of the original trial, we did not have access to the original survival data and, as a result, the Kaplan-Meier curves of CONFIRM were drawn based on the reported risk table which lacked detailed timepoints [15]. Having access to the underlying target trial data when emulating a trial would have been preferred.

Nevertheless, this study highlights how target trial emulations can bridge the gap between evidence from randomized controlled trials and from real-world patient data. Expanding trial findings to a more heterogeneous and real-world population offers insights that extend beyond the limits of traditional trials. More importantly, by focusing on a subgroup absent from randomized trials, e.g., the relapse-free patients, evidence can be generated directly applicable to underrepresented patients, supporting more inclusive healthcare decisions.

In conclusion, our study sheds light on the difference in effect estimates in trial-like populations and real-world populations, where the latter includes subgroups previously excluded from the CONFIRM target trial. Although the non-concurrency bias between the GA patients and DMF patients threatens validity of the presented estimates and therefore hinders clinical interpretation of the results, our approach still presents novel methodological insights in the form of the two-step approach using the TTE framework. This study can therefore be used as a guiding example for future observational studies that wish to incorporate the TTE framework, as well as embrace the wider variety in the patient population often ignored in experimental settings. The first step in our two-step approach thereby provides an indication that the correct methodology was applied by replicating trial results, while in the second step, the added value of the observational data is embraced.

## Supplementary Information

Below is the link to the electronic supplementary material.

Supplementary Material 1
