# Supplementary material for: A target trial emulation of the CONFIRM study with an extension to subgroups: an example for relapsing-remitting multiple sclerosis
Source: Eur J Epidemiol. 2026 Mar 18;41(7):841–53. doi: 10.1007/s10654-026-01379-1 (PMC13572341; doi:10.1007/s10654-026-01379-1)
Supplement: Supplementary file 1 [file 10654_2026_1379_MOESM1_ESM.docx]

Supplementary Information for
A Target Trial Emulation of the CONFIRM Study with an Extension to Subgroups: an Example for Relapsing-Remitting Multiple Sclerosis

**Corresponding author**: Stefan Verweij. Mail: [stefan.verweij@rug.nl](mailto:stefan.verweij@rug.nl). Tel: <+31646880454>.

# Table of contents

**Supplementary Figures**

Figure S1. Love plot of weighted population vs. unweighted population	p.2

Figure S2. Overlap of treatment initiation between DMF and GA cohort (pre- & post 2014)	p.3

Figure S3. Love plot of discontinued population vs. completed population	p.4

Figure S4. Kaplan-Meier curve for the probability of experiencing a first relapse (DMF)	p.5

Figure S5. Kaplan-Meier curve for the probability of experiencing a first relapse (GA)	p.6

Figure S6. Adjusted Kaplan-Meier curve for the probability of experiencing a first relapse (DMF vs. GA)	p.7

Figure S7. Adjusted Kaplan-Meier curve for the probability of experiencing a first relapse (DMF)	p.8

Figure S8. Adjusted Kaplan-Meier curve for the probability of experiencing a first relapse (GA)	p.9

Figure S9. Multicollinearity of additional covariates for the negative binomial model	p.10

Figure S10. Multicollinearity of additional covariates for the Cox proportional hazards model	p.10

Figure S11. Love plot of subgroup analysis with pre- and post-2012 GA subgroup	p.11

Figure S12. Subgroup analysis pre and post 2012 GA initiation (HR)	p.12

Figure S13. Subgroup analysis pre and post 2012 GA initiation (ARR ratio)	p.13

Figure S14. Assessing proportionality of hazards: Schoenfeld residuals per calendar year	p.14

**Supplementary Tables**

Table S1. List of first- and secondline disease modifying therapies	p.15

Table S2. Baseline characteristics for the overlap population	p.16

Table S3. Sample sizes for each scenario	p.17

Table S4. Reasons for censoring	p.18

Table S5. Baseline characteristics for the censoring population	p.20

Table S6. Baseline characteristics for the population that completed treatment	p.21

Table S7. Outcome table – crude ITT analysis	p.22

Table S8. Outcome table – PP analysis	p.23

Table S9. Outcome table – sensitivity analysis 1 – Poisson regression	p.23

Table S10. Outcome table – sensitivity analysis 2 – Increased pre-baseline SPMS conversion window	p.24

Table S11. Outcome table – sensitivity analysis 3 – G-formula	p.24

Table S12. Outcome table – sensitivity analysis 4 – Reduced baseline EDSS window	p.25

Table S13. Outcome table – sensitivity analysis 5 – Physician-confirmed relapses	p.26

Table S14. Outcome table – sensitivity analysis 6 – Additional covariates	p.27

Table S15. Baseline characteristics for the pre- and post-2012 GA population	p.29

Table S16. Outcome table – subgroup analysis – Pre and post 2012 GA subgroup	p.31

**Appendices**

Appendix 1. Rationale for selecting CONFIRM as target trial	p.33

Appendix 2. TARGET checklist	p.34

Appendix 3. Rationale for not including MRI	p.35

# Supplementary Figures

## Figure S1. Love plot of weighted population vs. unweighted population

A) For the strict trial-like population. Standardized Mean Differences of the covariates between the DMF and GA arms.

B) For the pragmatic population. Standardized Mean Differences of the covariates between the DMF and GA arms.

## Figure S2. Overlap of treatment initiation between DMF and GA cohort (pre-post 2014)

SI Figure 1. Period of treatment initiation of DMF versus GA. Almost all initiations of GA occurred prior to 2014, while all initiations of DMF occurred post 2014, in both (A) the strict scenario, and (B) the pragmatic scenario. DMF was authorized in the European Union in 2014. DMF=dimethyl fumarate, GA=glatiramer acetate.

## Figure S3. Love plot of discontinued population vs. completed population

A) For the strict trial-like population. Standardized Mean Differences of the covariates between the DMF and GA arms.

B) For the pragmatic population. Standardized Mean Differences of the covariates between the DMF and GA arms.

## Figure S4. Kaplan-Meier curve for the probability of experiencing a first relapse (DMF)

SI Figure 2. Kaplan-Meier curves comparing probability of experiencing a relapse in the DMF cohorts for the nine scenarios over 96 weeks. Unadjusted probability curve taken from the original CONFIRM trial. DMF=dimethyl fumarate.

## Figure S5. Kaplan-Meier curve for the probability of experiencing a first relapse (GA)

SI Figure 3. Kaplan-Meier curves comparing probability of experiencing a relapse in the GA cohorts for the nine scenarios over 96 weeks. Unadjusted probability curve taken from the original CONFIRM trial. GA=glatiramer acetate.

## Figure S6. Adjusted Kaplan-Meier curve for the probability of experiencing a first relapse (DMF vs. GA)

SI Figure 4. Adjusted Kaplan-Meier curves comparing probability of experiencing a relapse between DMF (dotted lines) and GA (solid lines) for the strict trial-emulation (blue) and the pragmatic scenario (red) over 96 weeks. Unadjusted probability curve taken from the original CONFIRM trial (green). DMF=dimethyl fumarate, GA=glatiramer acetate.

## Figure S7. Adjusted Kaplan-Meier curve for the probability of experiencing a first relapse (DMF)

SI Figure 5. Adjusted Kaplan-Meier curves comparing probability of experiencing a relapse in the DMF cohorts for the eight scenarios over 96 weeks. Unadjusted probability curve taken from the original CONFIRM trial. DMF=dimethyl fumarate.

## Figure S8. Adjusted Kaplan-Meier curve for the probability of experiencing a first relapse (GA)

 SI Figure 6. Adjusted Kaplan-Meier curves comparing probability of experiencing a relapse in the GA cohorts for the eight scenarios over 96 weeks. Unadjusted probability curve taken from the original CONFIRM trial. GA=glatiramer acetate.

## Figure S9 Multicollinearity of additional covariates for the negative binomial model

SI Figure 7. Generalized variance inflation factors (GVIF) for each of the covariates adjusted for in the negative binomial regression model for the sensitivity analysis with extra covariates. A GVIF of 1 indicates no multicollinearity, and above 2.5 indicates substantial multicollinearity. 1st DMT = history of firstline DMT usage, 2nd DMT = history of secondline DMT usage, ARR=annualized relapse rate, Calendar Y = pre- or post-authorization of dimethyl fumarate (2014) and EDSS=Expanded Disability Status Scale.

## Figure S10. Multicollinearity of additional covariates for the Cox proportional hazards model

SI Figure 8. Generalized variance inflation factors (GVIF) for each of the covariates adjusted for in the Cox proportional hazards model for the sensitivity analysis with extra covariates. A GVIF of 1 indicates no multicollinearity, and above 2.5 indicates substantial multicollinearity. 1st DMT = history of firstline DMT usage, 2nd DMT = history of secondline DMT usage, ARR=annualized relapse rate, Calendar Y = pre- or post-authorization of dimethyl fumarate (2014) and EDSS=Expanded Disability Status Scale.

## Figure S11. Love plot of subgroup analysis with pre- and post-2012 GA subgroup

Love plot of the subgroup with GA patients initiating treatment prior to 2012 versus the subgroup with GA patients initiating treatment after 2012.

A) For the strict trial-like population. Standardized Mean Differences of the covariates between the DMF and GA arms.

B) For the pragmatic population. Standardized Mean Differences of the covariates between the DMF and GA arms.

## Figure S12. Subgroup analysis pre and post 2012 GA initiation (HR ratio)

Subgroup analysis where the GA arm ($n_{srict scenario}=177$ and $n_{pragmatic scenario}=759)$ is stratified on their treatment initiation year to visualize the effect of possible calendar bias in estimating the HR between DMF and GA. An HR ratio below 1 favors DMF, while a ratio above 1 favors GA. **A)** The GA arm only contains subjects that initiated treatment prior to 2012 ($n_{strict scenario}=147$ and $n_{pragmatic scenario}=523$). **B)** The GA arm only contains subjects that initiated treatment after 2012 ($n_{strict scenario}=30$ and $n_{pragmatic scenario}=236$). DMF=dimethyl fumarate, EDSS=Expanded Disability Status Scale and GA=glatiramer acetate, HR=hazard ratio.

## Figure S13. Subgroup analysis pre and post 2012 GA initiation (ARR ratio)

Subgroup analysis where the GA arm ($n_{srict scenario}=177$ and $n_{pragmatic scenario}=759)$ is stratified on their treatment initiation year to visualize the effect of possible calendar bias in estimating the ARR ratio between DMF and GA. An ARR ratio below 1 favors DMF, while a ratio above 1 favors GA. **A)** The GA arm only contains subjects that initiated treatment prior to 2012 ($n_{strict scenario}=147$ and $n_{pragmatic scenario}=523$). **B)** The GA arm only contains subjects that initiated treatment after 2012 ($n_{strict scenario}=30$ and $n_{pragmatic scenario}=236$). ARR=annualized relapse rate, DMF=dimethyl fumarate, EDSS=Expanded Disability Status Scale and GA=glatiramer acetate.

## Figure S14. Assessing proportionality of hazards

Schoenfeld residuals of the treatment effect obtained from the Cox PH model plotted against the calendar year of treatment initiation, for **A)** the strict scenario and **B)** the pragmatic scenario.

# Supplementary Tables

## Table S1. List of first- and secondline disease modifying therapies

| **Firstline DMTs** | **Secondline DMTs** |
| --- | --- |
| Dimethyl fumarate | Alemtuzumab |
| Diroximel fumarate | Cladribine |
| Glatiramer acetate | Fingolimod |
| Interferon β-1a (Avonex) | Mitoxantrone |
| Interferon β-1a (Rebif) | Natalizumab |
| Interferon β-1b (Betaferon) | Ocrelizumab |
| Interferon β-1b (Extavia) | Ofatumumab |
| Peginterferon | Ozanimod |
| Ponesimod | Siponimod |
| Rituximab |  |
| Teriflunomide |  |

DMT = disease modifying therapy

## Table S2. Baseline characteristics for the overlap population

| ITT | *Strict trial-emulation* | | | *Pragmatic scenario* | | |
| --- | --- | --- | --- | --- | --- | --- |
| **Eligibility criteria** | **Age** | **EDSS** | **ARR** | **Age** | **EDSS** | **ARR** |
|  | 18-55 | ≤5 | ≥1 |  |  |  |
|  | **DMF** | **GA** | **p** | **DMF** | **GA** | **p** |
| **n** | 111 | 177 |  | 1072 | 759 |  |
| **Person-years** | 204 | 326 |  | 1974 | 1397 |  |
| **age (mean (SD))** | 34.1 (8.0) | 35.6 (8.2) | 0.09 | 38.5 (10.7) | 40 (10.7) | **0.003** |
| **Sex (%)*** |  |  | 0.48 |  |  | **0.001** |
| **Female (%)** | 81 (73) | 137 (77) |  | 754 (70) | 585 (77) |  |
| **Male (%)** | 30 (27) | 40 (23) |  | 318 (30) | 174 (23) |  |
| **EDSS at baseline  (mean (SD))** | 1.7 (1.1) | 1.8 (1.3) | 0.44 | 1.6 (1.3) | 1.9 (1.5) | **<0.001** |
| **Number of relapses in previous 12 months (mean (SD))** | 1.2 (0.5) | 1.4 (0.7) | 0.06 | 0.1 (0.4) | 0.3 (0.6) | **<0.001** |
| **Number of relapses in previous 12 months (%)*** |  |  | 0.09 |  |  | **<0.001** |
| **0** | 0 (0) | 0 (0) |  | 955 (89) | 575 (76) |  |
| **1** | 87 (78) | 132 (74) |  | 93 (9) | 140 (18) |  |
| **2** | 22 (20) | 29 (16) |  | 21 (2) | 32 (4) |  |
| **3** | 2 (2) | 13 (7) |  | 2 (0) | 10 (1) |  |
| **4** | 0 (0) | 4 (2) |  | 0 (0) | 2 (0) |  |
| **Number of pre-baseline DMT treatments (%)*** |  |  | **0.008** |  |  | **<0.001** |
| **0** | 88 (79) | 110 (62) |  | 745 (70) | 450 (59) |  |
| **1** | 18 (16) | 54 (31) |  | 227 (21) | 261 (34) |  |
| **2** | 4 (4) | 13 (7) |  | 77 (7) | 46 (6) |  |
| **3** | 1 (1) | 0 (0) |  | 18 (2) | 2 (0) |  |
| **4** |  |  |  | 5 (1) | 0 (0) |  |
| **Of which firstline therapies (%)*** |  |  | **0.003** |  |  | **<0.001** |
| **0** | 90 (81) | 111 (63) |  | 756 (70) | 457 (60) |  |
| **1** | 17 (15) | 54 (31) |  | 255 (24) | 270 (36) |  |
| **2** | 4 (4) | 13 (7) |  | 55 (5) | 31 (4) |  |
| **3** | 0 (0) | 0 (0) |  | 6 (1) | 2 (0) |  |
| **Of which secondline therapies (%)*** |  |  | 0.44 |  |  | **0.004** |
| **0** | 108 (97) | 174 (98) |  | 1017 (95) | 739 (97) |  |
| **1** | 2 (2) | 3 (2) |  | 38 (4) | 19 (2) |  |
| **2** | 1 (1) | 0 (0) |  | 16 (2) | 1 (0) |  |
| **Years since diagnosis (SD)** | 1.5 (3.8) | 2.5 (3.9) | 0.05 | 2.9 (5.0) | 3.6 (5.4) | **0.004** |
| * Due to rounding of the pooled estimates, the percentages may not add up to exactly 100%, and the summation of categorical variables may not be equal to n. | | | | | | |

## Table S3. Sample sizes for each scenario

|  | *Eligibility criteria* | | | *n* | | |
| --- | --- | --- | --- | --- | --- | --- |
| **Scenario** | **Age** | **EDSS** | **ARR** | **DMF** | **GA** | **Total** |
| CONFIRM trial | 18-55 | ≤5 | ≥1 | 359 | 350 | 709 |
| Strict trial-emulation | 18-55 | ≤5 | ≥1 | 111 | 177 | 288 |
| Pragmatic scenario |  |  |  | 1072 | 759 | 1831 |
| Age restricted | 18-55 |  |  | 994 | 681 | 1675 |
| EDSS restricted |  | ≤5 |  | 1055 | 739 | 1794 |
| ARR restricted |  |  | ≥1 | 115 | 191 | 306 |
| Age & EDSS restricted | 18-55 | ≤5 |  | 982 | 667 | 1649 |
| Age & ARR restricted | 18-55 |  | ≥1 | 113 | 181 | 294 |
| EDSS & ARR restricted |  | ≤5 | ≥1 | 113 | 187 | 300 |
| Pre-baseline ARR=0 |  |  | 0^†^ | 957 | 568 | 1525 |
| Age >55 | >55^*^ |  |  | 2 | 10 | 12 |
| EDSS >5 |  | >5^‡^ |  | 2 | 4 | 6 |

Sample sizes of each scenario, and which eligibility criteria was applied: 1) Restricting by age 18-55 years at baseline, 2) EDSS between 0 and 5 at baseline and 3) At least 1 relapse in the year prior to baseline (pre-baseline ARR≥1). ARR=annualized relapse rate, DMF=dimethyl fumarate, EDSS=Expanded Disability Status Scale, GA=glatiramer acetate.
^†^The criteria was reversed in this scenario to only focus at the subgroup with inactive disease, i.e., zero relapses in the year prior to baseline (pre-baseline ARR=0).
^*^The criteria was reversed in this scenario to only focus at the subgroup aged 56 years or older. SMSreg does not contain paediatric patients. ^‡^The criteria was reversed in this scenario to only focus at the subgroup with an EDSS score above 5.

## Table S4. Reasons for censoring

A) **Reasons for censoring in the main analysis:**

| **Strict scenario** | ITT analysis | | PP analysis | |
| --- | --- | --- | --- | --- |
| Censoring reason | Number of DMF patients (%) | Number of GA patients (%) | Number of DMF patients (%) | Number of GA patients (%) |
| Death | 0 | 0 | 0 | 0 |
| End of study duration  (96 weeks) | 111 (100) | 177 (100) | 44 (40) | 90 (51) |
| End of data collection | 0 | 0 | 0 | 0 |
| Loss to follow-up | 0 | 0 | 0 | 0 |
| Treatment discontinuation^*^ | *NA* | *NA* | 67 (60) | 88 (49) |

*^*Discontinuation is defined by those who discontinue treatment or switch to another treatment^*

| **Pragmatic scenario** | ITT analysis | | PP analysis | |
| --- | --- | --- | --- | --- |
| Censoring reason | Number of DMF patients (%) | Number of GA patients (%) | Number of DMF patients (%) | Number of GA patients (%) |
| Death | 0 | 0 | 0 | 2 (0) |
| End of study duration  (96 weeks) | 1068 (100) | 757 (100) | 588 (55) | 437 (58) |
| End of data collection | 4 (0) | 2 (0) | 4 (0) | 2 (0) |
| Loss to follow-up | 0 | 0 | 1 (0) | 2 (0) |
| Treatment discontinuation^*^ | *NA* | *NA* | 479 (45) | 316 (42) |

*^*Discontinuation is defined by those who discontinue treatment or switch to another treatment^*

B) **Reasons for censoring in the subgroup with GA patients initiating treatment before 2012:**

| **Strict scenario** | ITT analysis | | PP analysis | |
| --- | --- | --- | --- | --- |
| Censoring reason | Number of DMF patients (%) | Number of pre 2012 GA patients (%) | Number of DMF patients (%) | Number of pre 2012 GA patients (%) |
| Death | 0 | 0 | 0 | 0 |
| End of study duration  (96 weeks) | 111 (100) | 147 (100) | 44 (40) | 83 (56) |
| End of data collection | 0 | 0 | 0 | 0 |
| Loss to follow-up | 0 | 0 | 0 | 0 |
| Treatment discontinuation^*^ | *NA* | *NA* | 67 (60) | 65 (44) |

*^*Discontinuation is defined by those who discontinue treatment or switch to another treatment^*

| **Pragmatic scenario** | ITT analysis | | PP analysis | |
| --- | --- | --- | --- | --- |
| Censoring reason | Number of DMF patients (%) | Number of pre 2012 GA patients (%) | Number of DMF patients (%) | Number of pre 2012 GA patients (%) |
| Death | 0 | 0 | 0 | 1 (0) |
| End of study duration (96 weeks) | 1068 (100) | 523 (100) | 588 (55) | 331 (63) |
| End of data collection | 4 (0) | 0 | 4 (0) | 0 |
| Loss to follow-up | 0 | 0 | 1 (0) | 2 (0) |
| Treatment discontinuation^*^ | *NA* | *NA* | 479 (45) | 189 (36) |

*^*Discontinuation is defined by those who discontinue treatment or switch to another treatment^*

C) **Reasons for censoring in the subgroup with GA patients initiating treatment after 2012:**

| **Strict scenario** | ITT analysis | | PP analysis | |
| --- | --- | --- | --- | --- |
| Censoring reason | Number of DMF patients | Number of post 2012 GA patients | Number of DMF patients | Number of post 2012 GA patients |
| Death | 0 | 0 | 0 | 0 |
| End of study duration  (96 weeks) | 111 (100) | 30 (100) | 44 (40) | 7 (23) |
| End of data collection | 0 | 0 | 0 | 0 |
| Loss to follow-up | 0 | 0 | 0 | 0 |
| Treatment discontinuation^*^ | *NA* | *NA* | 67 (60) | 23 (77) |

*^*Discontinuation is defined by those who discontinue treatment or switch to another treatment^*

| **Pragmatic scenario** | ITT analysis | | PP analysis | |
| --- | --- | --- | --- | --- |
| Censoring reason | Number of DMF patients | Number of post 2012 GA patients | Number of DMF patients | Number of post 2012 GA patients |
| Death | 0 | 0 | 0 | 0 |
| End of study duration  (96 weeks) | 1068 (100) | 234 (99) | 588 (55) | 106 (45) |
| End of data collection | 4 (0) | 2 (1) | 4 (0) | 2 (1) |
| Loss to follow-up | 0 | 0 | 1 (0) | 0 |
| Treatment discontinuation^*^ | *NA* | *NA* | 479 (45) | 127 (54) |

*^*Discontinuation is defined by those who discontinue treatment or switch to another treatment^*

## Table S5. Baseline characteristics for the censoring population

The censoring population consists of those patients who discontinued treatment before study end (96 weeks) in the per-protocol analysis (SI Table S4). Discontinuation was defined by treatment stop or switching to a new treatment.

| PP | *Strict trial-emulation* | | | *Pragmatic scenario* | | |
| --- | --- | --- | --- | --- | --- | --- |
| **Eligibility criteria** | **Age** | **EDSS** | **ARR** | **Age** | **EDSS** | **ARR** |
|  | **✓** | **✓** | **✓** |  |  |  |
|  | **DMF** | **GA** | **p** | **DMF** | **GA** | **p** |
| **n** | 67 | 88 |  | 479 | 316 |  |
| **Person-years** | 51 | 62 |  | 369 | 223 |  |
| **age (mean (SD))** | 33.9 (8.1) | 34.9 (8.6) | 0.47 | 37.0 (10.7) | 37.9 (10.9) | 0.29 |
| **Sex (%)*** |  |  | 0.29 |  |  | 0.13 |
| **Female (%)** | 49 (73) | 71 (81) |  | 360 (75) | 253 (80 |  |
| **Male (%)** | 18 (27) | 16 (18) |  | 119 (25) | 63 (20) |  |
| **EDSS at baseline  (mean (SD))** | 1.7 (1.2) | 1.8 (1.2) | 0.75 | 1.6 (1.3) | 1.9 (1.5) | **0.002** |
| **Number of relapses in previous 12 months (mean (SD))** | 1.3 (0.5) | 1.5 (0.8) | **0.04** | 0.2 (0.5) | 0.4 (0.8) | **<0.001** |
| **Number of relapses in previous 12 months (%)*** |  |  | 0.09 |  |  | **<0.001** |
| **0** | 49 (73) | 57 (65) |  | 408 (85) | 224 (71) |  |
| **1** | 17 (25) | 20 (23) |  | 52 (11) | 59 (19) |  |
| **2** | 1 (1) | 9 (10) |  | 17 (4) | 22 (7) |  |
| **3** | 0 (0) | 2 (2) |  | 1 (0) | 9 (3) |  |
| **4** | 0 (0) | 0 (0) |  | 1 (0) | 2 (1) |  |
| **Number of pre-baseline DMT treatments (%)*** |  |  | 0.29 |  |  | **<0.001** |
| **0** | 53 (79) | 53 (60) |  | 336 (70) | 176 (56) |  |
| **1** | 11 (16) | 28 (32) |  | 98 (20) | 114 (36) |  |
| **2** | 3 (4) | 7 (8) |  | 33 (7) | 24 (8) |  |
| **3** |  |  |  | 10 (2) | 2 (1) |  |
| **4** |  |  |  | 2 (0) | 0 (0) |  |
| **Of which firstline therapies (%)*** |  |  | **0.02** |  |  | **<0.001** |
| **0** | 55 (82) | 54 (61) |  | 343 (72) | 179 (57) |  |
| **1** | 9 (13) | 28 (32 |  | 110 (23) | 120 (38) |  |
| **2** | 3 (4) | 6 (7) |  | 22 (5) | 15 (5) |  |
| **3** | 0 (0) | 0 (0) |  | 4 (1) | 2 (1) |  |
| **Of which secondline therapies (%)*** |  |  | 1 |  |  | **0.04** |
| **0** | 65 (97) | 86 (98) |  | 453 (95) | 304 (96) |  |
| **1** | 2 (3) | 2 (2) |  | 16 (3) | 12 (4) |  |
| **2** | 0 (0) | 0 (0) |  | 10 (2) | 0 (0) |  |
| **Years since diagnosis (SD)** | 1.2 (3.5) | 2.2 (4.1) | 0.11 | 2.6 (4.7) | 3.4 (5.1) | **0.02** |
| *Due to rounding of the pooled estimates after imputation, the percentages may not add up to exactly 100%, and the summation of categorical variables may not be equal to *n*. | | | | | | |

## Table S6. Baseline characteristics for the population that completed treatment

| PP | *Strict trial-emulation* | | | *Pragmatic scenario* | | |
| --- | --- | --- | --- | --- | --- | --- |
| **Eligibility criteria** | **Age** | **EDSS** | **ARR** | **Age** | **EDSS** | **ARR** |
|  | **✓** | **✓** | **✓** |  |  |  |
|  | **DMF** | **GA** | **p** | **DMF** | **GA** | **p** |
| **n** | 44 | 90 |  | 593 | 443 |  |
| **Person-years** | 81 | 166 |  | 1092 | 812 |  |
| **age (mean (SD))** | 35.3 (8.2) | 37.2 (7.9) | 0.19 | 39.1 (10.6) | 41.1 (10.5) | **0.002** |
| **Sex (%)*** |  |  | 1 |  |  | **0.004** |
| **Female (%)** | 32 (73) | 65 72) |  | 394 (66) | 332 (75) |  |
| **Male (%)** | 12 (28) | 25 (28) |  | 199 (34) | 111 (25) |  |
| **EDSS at baseline  (mean (SD))** | 1.7 (1.1) | 2.0 (1.4) | 0.29 | 1.5 (1.3) | 1.8 (1.4) | **<0.001** |
| **Number of relapses in previous 12 months (mean (SD))** | 1.2 (0.5) | 1.4 (0.9) | 0.15 | 0.1 (0.4) | 0.3 (0.7) | **<0.001** |
| **Number of relapses in previous 12 months (%)*** |  |  | 0.34 |  |  | **<0.001** |
| **0** | 35 (80) | 68 (76) |  | 549 (93) | 344 (78) |  |
| **1** | 7 (16) | 10 (11) |  | 35 (6) | 73 (16) |  |
| **2** | 2 (5) | 7 (8) |  | 7 (1) | 14 (3) |  |
| **3** | 0 (0) | 5 (6) |  | 2 (0) | 7 (2) |  |
| **4** | 0 (0) | 0 (0) |  | 0 (0) | 5 (1) |  |
| **Number of pre-baseline DMT treatments (%)*** |  |  | 1 |  |  | **<0.001** |
| **0** | 34 (77) | 57 (63) |  | 412 (69) | 276 (62) |  |
| **1** | 8 (18) | 27 (30) |  | 127 (21) | 145 (33) |  |
| **2** | 1 (2) | 6 (7) |  | 44 (7) | 22 (5) |  |
| **3** | 1 (2) | 0 (0) |  | 7 (1) | 0 (0) |  |
| **4** | 0 (0) | 0 (0) |  | 3 (1) | 0 (0) |  |
| **Of which firstline therapies (%)*** |  |  | 0.26 |  |  | **<0.001** |
| **0** | 34 (77) | 57 (63) |  | 415 (70) | 279 (63) |  |
| **1** | 9 (20) | 28 (30) |  | 143 (24) | 148 (33) |  |
| **2** | 1 (2) | 5 (6) |  | 33 (6) | 16 (4) |  |
| **3** | 0 (0) | 0 (0) |  | 2 (0) | 0 (0) |  |
| **Of which secondline therapies (%)*** |  |  | 0.28 |  |  | **0.05** |
| **0** | 43 (98) | 89 (99) |  | 566 (95) | 435 (98) |  |
| **1** | 0 (0) | 1 (1) |  | 21 (4) | 7 (2) |  |
| **2** | 1 (2) | 0 (0) |  | 6 (1) | 1 (0) |  |
| **Years since diagnosis (SD)** | 1.2 (3.5) | 2.4 (3.7) | 0.09 | 3.0 (5.2) | 3.6 (5.5) | 0.11 |
| *Due to rounding of the pooled estimates after imputation, the percentages may not add up to exactly 100%, and the summation of categorical variables may not be equal to *n*. | | | | | | |

## Table S7. Outcome table – crude ITT analysis

| **ITT** | **Strict scenario** | | | **Pragmatic scenario** | | | **EDSS & ARR restricted** | | | **Age & ARR restricted** | | | **Age & EDSS restricted** | | | **pre-baseline ARRs restricted** | | | **Age restricted** | | | **EDSS restricted** | | | **pre-baseline ARR=0** | | |
| --- | --- | --- | --- | --- | --- | --- | --- | --- | --- | --- | --- | --- | --- | --- | --- | --- | --- | --- | --- | --- | --- | --- | --- | --- | --- | --- | --- |
| **Eligibility criteria**^§^ | **Age** | **EDSS** | **ARR** | **Age** | **EDSS** | **ARR** | **Age** | **EDSS** | **ARR** | **Age** | **EDSS** | **ARR** | **Age** | **EDSS** | **ARR** | **Age** | **EDSS** | **ARR** | **Age** | **EDSS** | **ARR** | **Age** | **EDSS** | **ARR** | **Age** | **EDSS** | **ARR** |
|  | **18-55** | **≤5** | **≥1** |  |  |  |  | **≤5** | **≥1** | **18-55** |  | **≥1** | **18-55** | **≤5** |  |  |  | **≥1** | **18-55** |  |  |  | **≤5** |  |  |  | **0^†^** |
| **n** | 288 | | | 1831 | | | 300 | | | 294 | | | 1649 | | | 306 | | | 1675 | | | 1794 | | | 1525 | | |
| **person-years** | 530 | | | 3371 | | | 552 | | | 541 | | | 3036 | | | 563 | | | 3084 | | | 3303 | | | 2808 | | |
| **ARR GA** | 1.04 [0.90;1.20] | | | 0.35 [0.30;0.41] | | | 1.01 [0.88;1.17] | | | 1.03 [0.89;1.19] | | | 0.37 [0.32;0.43] | | | 1.00 [0.87;1.16] | | | 0.37 [0.32;0.43] | | | 0.36 [0.31;0.41] | | | 0.13 [0.10;0.17] | | |
| **ARR DMF** | 0.76 [0.61;0.95] | | | 0.14 [0.12;0.17] | | | 0.78 [0.63;0.96] | | | 0.79 [0.64;0.97] | | | 0.14 [0.12;0.17] | | | 0.80 [0.65;0.98] | | | 0.15 [0.12;0.18] | | | 0.14 [0.12;0.17] | | | 0.06 [0.05;0.08] | | |
| **ARRR** | 0.74 [0.57;0.96] | | | 0.40 [0.32;0.51] | | | 0.77 [0.60;0.99] | | | 0.77 [0.60;0.99] | | | 0.39 [0.31;0.49] | | | 0.80 [0.62;1.02] | | | 0.40 [0.32;0.51] | | | 0.29 [0.31;0.49] | | | 0.48 [0.33;0.71] | | |
| **Proportion relapsed after 2 years (GA)** | 68% | | | 28% | | | 68% | | | 68% | | | 29% | | | 68% | | | 29% | | | 29% | | | 13% | | |
| **Proportion relapsed after 2 years (DMF)** | 69% | | | 11% | | | 69% | | | 69% | | | 12% | | | 69% | | | 12% | | | 12% | | | 5% | | |
| **HR** | 1.05 [0.83;1.34] | | | 0.41 [0.34;0.48] | | | 1.08 [0.85;1.37] | | | 1.06 [0.84;1.35] | | | 0.41 [0.34;0.49] | | | 1.09 [0.86;1.37] | | | 0.41 [0.34;0.50] | | | 0.40 [0.33;0.48] | | | 0.38 [0.29;0.50] | | |
| ^§^The three eligibility criteria are 1) age at baseline between 18 and 55 year, 2) an EDSS score at baseline between 0 and 5, and 3) having had at least one relapse in the year prior to baseline. ^†^This is an adjusted eligibility criteria where a patient had no relapses in the year prior to baseline as opposed to CONFIRM’s criteria of having at least one relapse in the year prior to baseline. | | | | | | | | | | | | | | | | | | | | | | | | | | | |

## Table S8. Outcome table – PP analysis

| **PP** | **CONFIRM** | | | **Strict scenario** | | | **Pragmatic  scenario** | | | **EDSS & ARR restricted** | | | **Age & ARR restricted** | | | **Age & EDSS restricted** | | | **pre-baseline ARRs restricted** | | | **Age  restricted** | | | **EDSS  restricted** | | | **pre-baseline ARR=0** | | | |
| --- | --- | --- | --- | --- | --- | --- | --- | --- | --- | --- | --- | --- | --- | --- | --- | --- | --- | --- | --- | --- | --- | --- | --- | --- | --- | --- | --- | --- | --- | --- | --- |
| **Eligibility criteria**^§^ | **Age** | **EDSS** | **ARR** | **Age** | **EDSS** | **ARR** | **Age** | **EDSS** | **ARR** | **Age** | **EDSS** | **ARR** | **Age** | **EDSS** | **ARR** | **Age** | **EDSS** | **ARR** | **Age** | **EDSS** | **ARR** | **Age** | **EDSS** | **ARR** | **Age** | **EDSS** | **ARR** | **Age** | **EDSS** | **ARR** |  |
|  | **18-55** | **≤5** | **≥1** | **18-55** | **≤5** | **≥1** |  |  |  |  | **≤5** | **≥1** | **18-55** |  | **≥1** | **18-55** | **≤5** |  |  |  | **≥1** | **18-55** |  |  |  | **≤5** |  |  |  | **0^†^** |  |
| **n** | 704 | | | 288 | | | 1831 | | | 300 | | | 294 | | | 1649 | | | 306 | | | 1675 | | | 1794 | | | 1525 | | | |
| **person-years** | 1175 | | | 380 | | | 2654 | | | 397 | | | 390 | | | 2392 | | | 407 | | | 2429 | | | 2598 | | | 2248 | | | |
| **ARR GA** | *na*^‡^ | | | 1.63 [0.36;7.32] | | | 0.40 [0.13;1.26] | | | 1.72 [0.42;6.94] | | | 1.64 [0.37;7.31] | | | 0.33 [0.095;1.26] | | | 1.73 [0.43;6.97] | | | 0.33 [0.09;1.27] | | | 0.38 [0.12;1.23] | | | 0.27 [0.04;1.89] | | | |
| **ARR DMF** | *na*^‡^ | | | 1.40 [0.33;6.01] | | | 0.24 [0.08;0.73] | | | 1.50 [0.39;5.72] | | | 1.48 [0.35;6.28] | | | 0.20 [0.05;0.73] | | | 1.58 [0.42;5.99] | | | 0.21 [0.06;0.76] | | | 0.22 [0.07;0.68] | | | 0.12 [0.02;0.77] | | | |
| **ARRR** | 0.76^*^ [0.51;1.12] | | | 0.86 [0.46;1.59] | | | 0.61 [0.35;1.07] | | | 0.87 [0.47;1.61] | | | 0.90 [0.49;1.64] | | | 0.60 [0.33;1.09] | | | 0.91 [0.50;1.67] | | | 0.63 [0.35;1.13] | | | 0.58 [0.33;1.03] | | | 0.45 [0.17;1.19] | | | |
| **Proportion relapsed after 2 years (GA)** | *na*^‡^ | | | 67% | | | 19% | | | 67% | | | 67% | | | 20% | | | 67% | | | 20% | | | 19% | | | 11% | | | |
| **Proportion relapsed after 2 years (DMF)** | *na*^‡^ | | | 66% | | | 12% | | | 66% | | | 67% | | | 12% | | | 66% | | | 12% | | | 12% | | | 4% | | | |
| **HR** | *na*^‡^ | | | 0.97 [0.73;1.29] | | | 0.55 [0.43;0.69] | | | 0.97 [0.73;1.28] | | | 0.98 [0.74;1.30] | | | 0.56 [0.44;0.71] | | | 0.98 [0.74;1.30] | | | 0.57 [0.45;0.73] | | | 0.53 [0.42;0.68] | | | 0.36 [0.25;0.52] | | | |
| ^§^The three eligibility criteria are 1) age at baseline between 18 and 55 year, 2) an EDSS score at baseline between 0 and 5, and 3) having had at least one relapse in the year prior to baseline. ^†^This is an adjusted eligibility criteria where a patient had no relapses in the year prior to baseline as opposed to CONFIRM’s criteria of having at least one relapse in the year prior to baseline. | | | | | | | | | | | | | | | | | | | | | | | | | | | | | | | |
| *The ARRR between DMF & GA is derived from the ARRR of DMF vs. placebo and the ARRR of GA vs. placebo, as originally reported in the CONFIRM trial. | | | | | | | | | | | | | | | | | | | | | | | | | | | | | | | |
| ^‡^Results were not reported in the CONFIRM trial | | | | | | | | | | | | | | | | | | | | | | | | | | | | | | | |

## Table S9. Outcome table – sensitivity analysis 1 – Poisson regression

| **ITT** | **Strict scenario** | | | **Pragmatic scenario** | | | **EDSS & ARR restricted** | | | **Age & ARR restricted** | | | **Age & EDSS restricted** | | | **pre-baseline ARRs restricted** | | | **Age restricted** | | | **EDSS restricted** | | | **pre-baseline ARR=0** | | |
| --- | --- | --- | --- | --- | --- | --- | --- | --- | --- | --- | --- | --- | --- | --- | --- | --- | --- | --- | --- | --- | --- | --- | --- | --- | --- | --- | --- |
| **Eligibility criteria**^§^ | **Age** | **EDSS** | **ARR** | **Age** | **EDSS** | **ARR** | **Age** | **EDSS** | **ARR** | **Age** | **EDSS** | **ARR** | **Age** | **EDSS** | **ARR** | **Age** | **EDSS** | **ARR** | **Age** | **EDSS** | **ARR** | **Age** | **EDSS** | **ARR** | **Age** | **EDSS** | **ARR** |
|  | **18-55** | **≤5** | **≥1** |  |  |  |  | **≤5** | **≥1** | **18-55** |  | **≥1** | **18-55** | **≤5** |  |  |  | **≥1** | **18-55** |  |  |  | **≤5** |  |  |  | **0^†^** |
| **n** | 288 | | | 1831 | | | 300 | | | 294 | | | 1649 | | | 306 | | | 1675 | | | 1794 | | | 1525 | | |
| **person-years** | 530 | | | 3371 | | | 552 | | | 541 | | | 3036 | | | 563 | | | 3084 | | | 3303 | | | 2808 | | |
| **ARR GA** | 288 | | | 0.39 [0.17;0.90] | | | 1.19 [0.36;3.90] | | | 1.17 [0.32;4.20] | | | 0.33 [0.12;0.87] | | | 1.18 [0.36;3.86] | | | 0.33 [0.13;0.87] | | | 0.37 [0.16;0.88] | | | 0.27 [0.07;1.07] | | |
| **ARR DMF** | 576 | | | 0.23 [0.10;0.51] | | | 0.91 [0.29;2.85] | | | 0.91 [0.26;3.16] | | | 0.19 [0.07;0.49] | | | 0.94 [0.30;2.92] | | | 0.20 [0.08;0.51] | | | 0.21 [0.09;0.49] | | | 0.13 [0.03;0.49] | | |
| **ARRR** | 1.17 [0.32;4.26] | | | 0.59 [0.38;0.91] | | | 0.76 [0.44;1.31] | | | 0.78 [0.46;1.33] | | | 0.58 [0.37;0.92] | | | 0.79 [0.47;1.34] | | | 0.61 [0.39;0.95] | | | 0.57 [0.36;0.89] | | | 0.48 [0.23;0.98] | | |
| ^§^The three eligibility criteria are 1) age at baseline between 18 and 55 year, 2) an EDSS score at baseline between 0 and 5, and 3) having had at least one relapse in the year prior to baseline. ^†^This is an adjusted eligibility criteria where a patient had no relapses in the year prior to baseline as opposed to CONFIRM’s criteria of having at least one relapse in the year prior to baseline. | | | | | | | | | | | | | | | | | | | | | | | | | | | |

## Table S10. Outcome table – sensitivity analysis 2 – Increased pre-baseline SPMS conversion window

| **ITT** | **Strict scenario** | | | **Pragmatic  scenario** | | | **EDSS & ARR restricted** | | | **Age & ARR restricted** | | | **Age & EDSS restricted** | | | **pre-baseline ARRs restricted** | | | **Age restricted** | | | **EDSS restricted** | | | **pre-baseline ARR=0** | | |
| --- | --- | --- | --- | --- | --- | --- | --- | --- | --- | --- | --- | --- | --- | --- | --- | --- | --- | --- | --- | --- | --- | --- | --- | --- | --- | --- | --- |
| **Eligibility criteria**^§^ | **Age** | **EDSS** | **ARR** | **Age** | **EDSS** | **ARR** | **Age** | **EDSS** | **ARR** | **Age** | **EDSS** | **ARR** | **Age** | **EDSS** | **ARR** | **Age** | **EDSS** | **ARR** | **Age** | **EDSS** | **ARR** | **Age** | **EDSS** | **ARR** | **Age** | **EDSS** | **ARR** |
|  | **18-55** | **≤5** | **≥1** |  |  |  |  | **≤5** | **≥1** | **18-55** |  | **≥1** | **18-55** | **≤5** |  |  |  | **≥1** | **18-55** |  |  |  | **≤5** |  |  |  | **0^†^** |
| **n** | 290 | | | 1852 | | | 302 | | | 296 | | | 1664 | | | 309 | | | 1693 | | | 1810 | | | 1543 | | |
| **person-years** | 534 | | | 3410 | | | 556 | | | 545 | | | 3064 | | | 569 | | | 3117 | | | 3332 | | | 2841 | | |
| **ARR GA** | 1.20 [0.33;4.36] | | | 0.35 [0.14;0.90] | | | 1.20 [0.36;3.96] | | | 1.19 [0.33;4.29] | | | 0.29 [0.10;0.87] | | | 1.19 [0.36;3.90] | | | 0.29 [0.10;0.87] | | | 0.34 [0.13;0.89] | | | 0.26 [0.05;1.24] | | |
| **ARR DMF** | 0.90 [0.26;3.16] | | | 0.20 [0.08;0.50] | | | 0.92 [0.29;2.90] | | | 0.93 [0.27;3.23] | | | 0.17 [0.06;0.49] | | | 0.94 [0.30;2.95] | | | 0.18 [0.06;0.50] | | | 0.19 [0.08;0.48] | | | 0.13 [0.03;0.56] | | |
| **ARRR** | 0.75 [0.44;1.29] | | | 0.58 [0.36;0.94] | | | 0.76 [0.44;1.31] | | | 0.78 [0.46;1.33] | | | 0.58 [0.35;0.95] | | | 0.79 [0.47;1.35] | | | 0.60 [0.37;0.98] | | | 0.56 [0.35;0.91] | | | 0.49 [0.22;1.09] | | |
| **Proportion relapsed after 2 years (GA)** | 67% | | | 19% | | | 66% | | | 66% | | | 20% | | | 66% | | | 19% | | | 19% | | | 12% | | |
| **Proportion relapsed after 2 years (DMF)** | 68% | | | 12% | | | 68% | | | 69% | | | 13% | | | 68% | | | 13% | | | 12% | | | 5% | | |
| **HR** | 1.05 [0.83;1.33] | | | 0.56 [0.46;0.67] | | | 1.05 [0.83;1.33] | | | 1.06 [0.84;1.34] | | | 0.58 [0.48;0.70] | | | 1.06 [0.84;1.33] | | | 0.59 [0.49;0.71] | | | 0.55 [0.46;0.66] | | | 0.38 [0.29;0.50] | | |
| ^§^The three eligibility criteria are 1) age at baseline between 18 and 55 year, 2) an EDSS score at baseline between 0 and 5, and 3) having had at least one relapse in the year prior to baseline. ^†^This is an adjusted eligibility criteria where a patient had no relapses in the year prior to baseline as opposed to CONFIRM’s criteria of having at least one relapse in the year prior to baseline. | | | | | | | | | | | | | | | | | | | | | | | | | | | |

## Table S11. Outcome table – sensitivity analysis 3 – g-formula

| **ITT** | **Strict scenario** | | | **Pragmatic scenario** | | | **EDSS & ARR restricted** | | | **Age & ARR restricted** | | | **Age & EDSS restricted** | | | **pre-baseline ARRs restricted** | | | **Age restricted** | | | **EDSS restricted** | | | **pre-baseline ARR=0** | | |
| --- | --- | --- | --- | --- | --- | --- | --- | --- | --- | --- | --- | --- | --- | --- | --- | --- | --- | --- | --- | --- | --- | --- | --- | --- | --- | --- | --- |
| **Eligibility criteria**^§^ | **Age** | **EDSS** | **ARR** | **Age** | **EDSS** | **ARR** | **Age** | **EDSS** | **ARR** | **Age** | **EDSS** | **ARR** | **Age** | **EDSS** | **ARR** | **Age** | **EDSS** | **ARR** | **Age** | **EDSS** | **ARR** | **Age** | **EDSS** | **ARR** | **Age** | **EDSS** | **ARR** |
|  | **18-55** | **≤5** | **≥1** |  |  |  |  | **≤5** | **≥1** | **18-55** |  | **≥1** | **18-55** | **≤5** |  |  |  | **≥1** | **18-55** |  |  |  | **≤5** |  |  |  | **0^†^** |
| **n** | 288 | | | 1831 | | | 300 | | | 294 | | | 1649 | | | 306 | | | 1675 | | | 1794 | | | 1525 | | |
| **person-years** | 530 | | | 3371 | | | 552 | | | 541 | | | 3036 | | | 563 | | | 3084 | | | 3303 | | | 2808 | | |
| **ARRR** | 0,76 [0.60;0.93] | | | 0,58 [0.44;0.70] | | | 0,78 [0.63;0.95] | | | 0,78 [0.62;0.96] | | | 0,59 [0.46;0.73] | | | 0,81 [0.64;0.99] | | | 0,60 [0.46;0.76] | | | 0,56 [0.44;0.70] | | | 0,48 [0.32;0.71] | | |
| **HR** | 1.02 [0.81;1.28] | | | 0.63 [0.55;0.74] | | | 1.04 [0.81;1.32] | | | 1.04 [0.81;1.31] | | | 0.65 [0.55;0.77] | | | 1.05 [0.83;1.33] | | | 0.66 [0.55;0.78] | | | 0.63 [0.55;0.74] | | | 0.41 [0.31;0.54] | | |
| ^§^The three eligibility criteria are 1) age at baseline between 18 and 55 year, 2) an EDSS score at baseline between 0 and 5, and 3) having had at least one relapse in the year prior to baseline. ^†^This is an adjusted eligibility criteria where a patient had no relapses in the year prior to baseline as opposed to CONFIRM’s criteria of having at least one relapse in the year prior to baseline. | | | | | | | | | | | | | | | | | | | | | | | | | | | |

## Table S12. Outcome table – sensitivity analysis 4 – Reduced baseline EDSS window

| **ITT** | **Strict scenario** | | | **Pragmatic  scenario** | | | **EDSS & ARR restricted** | | | **Age & ARR restricted** | | | **Age & EDSS restricted** | | | **pre-baseline ARRs restricted** | | | **Age restricted** | | | **EDSS restricted** | | | **pre-baseline  ARR=0** | | |
| --- | --- | --- | --- | --- | --- | --- | --- | --- | --- | --- | --- | --- | --- | --- | --- | --- | --- | --- | --- | --- | --- | --- | --- | --- | --- | --- | --- |
| **Eligibility criteria**^§^ | **Age** | **EDSS** | **ARR** | **Age** | **EDSS** | **ARR** | **Age** | **EDSS** | **ARR** | **Age** | **EDSS** | **ARR** | **Age** | **EDSS** | **ARR** | **Age** | **EDSS** | **ARR** | **Age** | **EDSS** | **ARR** | **Age** | **EDSS** | **ARR** | **Age** | **EDSS** | **ARR** |
|  | **18-55** | **≤5** | **≥1** |  |  |  |  | **≤5** | **≥1** | **18-55** |  | **≥1** | **18-55** | **≤5** |  |  |  | **≥1** | **18-55** |  |  |  | **≤5** |  |  |  | **0^†^** |
| **n** | 264 | | | 1631 | | | 274 | | | 270 | | | 1478 | | | 280 | | | 1500 | | | 1597 | | | 1351 | | |
| **person-years** | 486 | | | 3003 | | | 504 | | | 497 | | | 2721 | | | 516 | | | 2762 | | | 2940 | | | 2487 | | |
| **ARR GA** | 1.18 [0.31;4.49] | | | 0.34 [0.13;0.91] | | | 1.16 [0.34;3.96] | | | 1.17 [0.31;4.44] | | | 0.28 [0.09;0.89] | | | 1.15 [0.34;3.93] | | | 0.28 [0.09;0.89] | | | 0.33 [0.12;0.89] | | | 0.24 [0.05;1.31] | | |
| **ARR DMF** | 0.90 [0.25;3.27] | | | 0.19 [0.08;0.50] | | | 0.89 [0.28;2.91] | | | 0.93 [0.26;3.36] | | | 0.16 [0.05;0.49] | | | 0.93 [0.29;2.99] | | | 0.17 [0.06;0.51] | | | 0.18 [0.07;0.47] | | | 0.11 [0.02;0.56] | | |
| **ARRR** | 0.76 [0.44;1.33] | | | 0.58 [0.35;0.95] | | | 0.77 [0.45;1.34] | | | 0.79 [0.46;1.37] | | | 0.57 [0.34;0.96] | | | 0.80 [0.34;1.38] | | | 0.60 [0.36;1.00] | | | 0.55 [0.33;0.91] | | | 0.46 [0.19;1.09] | | |
| **Proportion relapsed after 2 years (GA)** | 69% | | | 20% | | | 69% | | | 68% | | | 20% | | | 68% | | | 20% | | | 20% | | | 13% | | |
| **Proportion relapsed after 2 years (DMF)** | 69% | | | 12% | | | 69% | | | 69% | | | 13% | | | 69% | | | 13% | | | 12% | | | 5% | | |
| **HR** | 1.01 [0.78;1.30] | | | 0.53 [0.43;0.64] | | | 1.01 [0.78;1.29] | | | 1.02 [0.79;1.31] | | | 0.54 [044;0.66] | | | 1.01 [0.79;1.30] | | | 0.55 [0.49;0.67] | | | 0.52 [0.43;0.63] | | | 0.33 [0.24;0.45] | | |
| ^§^The three eligibility criteria are 1) age at baseline between 18 and 55 year, 2) an EDSS score at baseline between 0 and 5, and 3) having had at least one relapse in the year prior to baseline. ^†^This is an adjusted eligibility criteria where a patient had no relapses in the year prior to baseline as opposed to CONFIRM’s criteria of having at least one relapse in the year prior to baseline. | | | | | | | | | | | | | | | | | | | | | | | | | | | |

## Table S13. Outcome table – sensitivity analysis 5 – Physician-confirmed relapses

| **ITT** | **Strict scenario** | | | **Pragmatic  scenario** | | | **EDSS & ARR restricted** | | | **Age & ARR restricted** | | | **Age & EDSS restricted** | | | **pre-baseline ARRs restricted** | | | **Age restricted** | | | **EDSS restricted** | | | **pre-baseline  ARR=0** | | |
| --- | --- | --- | --- | --- | --- | --- | --- | --- | --- | --- | --- | --- | --- | --- | --- | --- | --- | --- | --- | --- | --- | --- | --- | --- | --- | --- | --- |
| **Eligibility criteria**^§^ | **Age** | **EDSS** | **ARR** | **Age** | **EDSS** | **ARR** | **Age** | **EDSS** | **ARR** | **Age** | **EDSS** | **ARR** | **Age** | **EDSS** | **ARR** | **Age** | **EDSS** | **ARR** | **Age** | **EDSS** | **ARR** | **Age** | **EDSS** | **ARR** | **Age** | **EDSS** | **ARR** |
|  | **18-55** | **≤5** | **≥1** |  |  |  |  | **≤5** | **≥1** | **18-55** |  | **≥1** | **18-55** | **≤5** |  |  |  | **≥1** | **18-55** |  |  |  | **≤5** |  |  |  | **0^†^** |
| **n** | 234 | | | 1723 | | | 243 | | | 239 | | | 1551 | | | 248 | | | 1574 | | | 1689 | | | 1475 | | |
| **person-years** | 431 | | | 3172 | | | 447 | | | 440 | | | 2856 | | | 457 | | | 2898 | | | 3110 | | | 2716 | | |
| **ARR GA** | 1.16 [0.28;4.86] | | | 0.31 [0.09;0.93] | | | 1.21 [0.31;4.76] | | | 1.17 [0.28;4.92] | | | 0.26 [0.07;0.95] | | | 1.23 [0.31;4.81] | | | 0.27 [0.07;0.98] | | | 0.29 [0.09;0.93] | | | 0.25 [0.04;1.67] | | |
| **ARR DMF** | 0.88 [0.22;3.59] | | | 0.18 [0.06;0.52] | | | 0.93 [0.25;3.49] | | | 0.91 [0.22;3.67] | | | 0.15 [0.04;0.53] | | | 0.96 [0.25;3.56] | | | 0.16 [0.05;0.56] | | | 0.16 [0.05;0.49] | | | 0.13 [0.02;0.74] | | |
| **ARRR** | 0.76 [0.41;1.41] | | | 0.57 [0.32;1.00] | | | 0.77 [0.42;1.42] | | | 0.77 [0.42;1.42] | | | 0.59 [0.33;1.06] | | | 0.78 [0.42;1.43] | | | 0.60 [0.34;1.07] | | | 0.56 [0.09;0.93] | | | 0.50 [0.19;1.28] | | |
| **Proportion relapsed after 2 years (GA)** | 58% | | | 13% | | | 58% | | | 58% | | | 14% | | | 58% | | | 14% | | | 13% | | | 9% | | |
| **Proportion relapsed after 2 years (DMF)** | 66% | | | 8% | | | 65% | | | 65% | | | 9% | | | 65% | | | 9% | | | 8% | | | 3% | | |
| **HR** | 1.23 [0.94;1.61] | | | 0.57 [0.46;0.71] | | | 1.23 [0.94;1.60] | | | 1.23 [0.94;1.60] | | | 0.59 [0.48;0.74] | | | 1.22 [0.94;1.59] | | | 0.60 [0.48;0.74] | | | 0.57 [0.46;0.70] | | | 0.38 [0.27;0.52] | | |
| ^§^The three eligibility criteria are 1) age at baseline between 18 and 55 year, 2) an EDSS score at baseline between 0 and 5, and 3) having had at least one relapse in the year prior to baseline. ^†^This is an adjusted eligibility criteria where a patient had no relapses in the year prior to baseline as opposed to CONFIRM’s criteria of having at least one relapse in the year prior to baseline. | | | | | | | | | | | | | | | | | | | | | | | | | | | |

## Table S14. Outcome table – sensitivity analysis 6 – Additional covariates

A) *A priori* defined sensitivity analysis with covariates: Age, baseline EDSS score, pre-index ARR, treatment history, calendar year and sex.

| **ITT** | **Strict scenario** | | | **Pragmatic scenario** | | | **EDSS & ARR restricted** | | | **Age & ARR restricted** | | | **Age & EDSS restricted** | | | **pre-baseline ARRs restricted** | | | **Age restricted** | | | **EDSS restricted** | | | **pre-baseline ARR=0** | | |
| --- | --- | --- | --- | --- | --- | --- | --- | --- | --- | --- | --- | --- | --- | --- | --- | --- | --- | --- | --- | --- | --- | --- | --- | --- | --- | --- | --- |
| **Eligibility criteria**^§^ | **Age** | **EDSS** | **ARR** | **Age** | **EDSS** | **ARR** | **Age** | **EDSS** | **ARR** | **Age** | **EDSS** | **ARR** | **Age** | **EDSS** | **ARR** | **Age** | **EDSS** | **ARR** | **Age** | **EDSS** | **ARR** | **Age** | **EDSS** | **ARR** | **Age** | **EDSS** | **ARR** |
|  | **18-55** | **≤5** | **≥1** |  |  |  |  | **≤5** | **≥1** | **18-55** |  | **≥1** | **18-55** | **≤5** |  |  |  | **≥1** | **18-55** |  |  |  | **≤5** |  |  |  | **0^†^** |
| **n** | 288 | | | 1831 | | | 300 | | | 294 | | | 1649 | | | 306 | | | 1675 | | | 1794 | | | 1525 | | |
| **person-years** | 530 | | | 3371 | | | 552 | | | 541 | | | 3036 | | | 563 | | | 3084 | | | 3303 | | | 2808 | | |
| **ARR GA** | 1.30 [0;inf] | | | 0.20 [0.00;16.31] | | | 1.27 [0;inf] | | | 1.20 [0.02;75.2] | | | 0.15 [0;inf] | | | 1.09 [0.03;43.00] | | | 0.16 [0.00;14.05] | | | 0.19 [0;inf] | | | 0.12 [0;inf] | | |
| **ARR DMF** | 0.90 [0;inf] | | | 0.10 [0.00;10.83] | | | 0.88 [0,inf] | | | 0.82 [0.01;64.77] | | | 0.08 [0;inf] | | | 0.75 [0.01;40.92] | | | 0.08 [0.00;9.32] | | | 0.10 [0;inf] | | | 0.05 [0;inf] | | |
| **ARRR** | 0.70 [0.12;3.95] | | | 0.52 [0.12;2.24] | | | 0.70 [0.12;3.96] | | | 0.69 [0.12;3.85] | | | 0.53 [0.12;2.25] | | | 0.69 [0.12;3.87] | | | 0.53 [0.12;2.28] | | | 0.52 [0.12;2.20] | | | 0.44 [0.04;4.79] | | |
| **Proportion relapsed after 2 years (GA)** | 60% | | | 14% | | | 60% | | | 65% | | | 14% | | | 65% | | | 15% | | | 14% | | | 9% | | |
| **Proportion relapsed after 2 years (DMF)** | 70% | | | 12% | | | 70% | | | 68% | | | 12% | | | 68% | | | 12% | | | 11% | | | 6% | | |
| **HR** | 1.40 [0.64;3.07] | | | 0.75 [0.47;1.19] | | | 1.40 [0.64;3.06] | | | 1.09 [0.60;1.98] | | | 0.78 [0.46;1.31] | | | 1.09 [0.61;1.97] | | | 0.78 [0.47;1.27] | | | 0.74 [0.45;1.20] | | | 0.59 [0.31;1.14] | | |
| ^§^The three eligibility criteria are 1) age at baseline between 18 and 55 year, 2) an EDSS score at baseline between 0 and 5, and 3) having had at least one relapse in the year prior to baseline. ^†^This is an adjusted eligibility criteria where a patient had no relapses in the year prior to baseline as opposed to CONFIRM’s criteria of having at least one relapse in the year prior to baseline. | | | | | | | | | | | | | | | | | | | | | | | | | | | |

B) *Post-hoc* defined sensitivity analysis with covariates: Age, baseline EDSS score, pre-index ARR and treatment history.

| **ITT** | **Strict scenario** | | | **Pragmatic scenario** | | | **EDSS & ARR restricted** | | | **Age & ARR restricted** | | | **Age & EDSS restricted** | | | **pre-index ARRs restricted** | | | **Age restricted** | | | | **EDSS restricted** | | | | **pre-index ARR=0** | | | |  |
| --- | --- | --- | --- | --- | --- | --- | --- | --- | --- | --- | --- | --- | --- | --- | --- | --- | --- | --- | --- | --- | --- | --- | --- | --- | --- | --- | --- | --- | --- | --- | --- |
| **Eligibility criteria**^§^ | **Age** | **EDSS** | **ARR** | **Age** | **EDSS** | **ARR** | **Age** | **EDSS** | **ARR** | **Age** | **EDSS** | **ARR** | **Age** | **EDSS** | **ARR** | **Age** | **EDSS** | **ARR** | | **Age** | **EDSS** | **ARR** | | **Age** | **EDSS** | **ARR** | | **Age** | **EDSS** | **ARR** | |
|  | **18-55** | **≤5** | **≥1** |  |  |  |  | **≤5** | **≥1** | **18-55** |  | **≥1** | **18-55** | **≤5** |  |  |  | **≥1** | | **18-55** |  |  | |  | **≤5** |  | |  |  | **0^†^** | |
| **n** | 288 | | | 1831 | | | 300 | | | 294 | | | 1649 | | | 306 | | | 1675 | | | | 1794 | | | | 1525 | | | |  |
| **person-years** | 530 | | | 3371 | | | 552 | | | 541 | | | 3036 | | | 563 | | | 3084 | | | | 3303 | | | | 2808 | | | |  |
| **ARR GA** | 1.17 [0.01;138.14] | | | 0.24 [0.02; 2.89] | | | 1.18 [0.01;134.24] | | | 1.15 [0.03;49.12] | | | 0.19 [0.01; 3.12] | | | 1.10 [0.04;31.63] | | | 0.19 [0.01;2.99] | | | | 0.23 [0.02;2.98] | | | | 0.14 [0.00;6.13] | | | |  |
| **ARR DMF** | 0.81 [0.01;54.35] | | | 0.13 [0.01;1.09] | | | 0.81 [0.01;52.67] | | | 0.79 [0.02;28.33] | | | 0.10 [0.01;1.15] | | | 0.76 [0.03;19.89] | | | 0.10 [0.01;1.17] | | | | 0.12 [0.01;1.07] | | | | 0.06 [0.00;1.77] | | | |  |
| **ARRR** | 0.70 [0.12;3.95] | | | 0.52 [0.13;2.09] | | | 0.70 [0.12;3.96] | | | 0.68 [0.16;2.85] | | | 0.52 [0.12;2.21] | | | 0.69 [0.17;2.86] | | | 0.54 [0.13;2.14] | | | | 0.51 [0.12;2.15] | | | | 0.43 [0.04;4.59] | | | |  |
| **Proportion relapsed after 2 years (GA)** | 60% | | | 13% | | | 60% | | | 66% | | | 13% | | | 65% | | | 14% | | | | 13% | | | | 10% | | | |  |
| **Proportion relapsed after 2 years (DMF)** | 72% | | | 11% | | | 72% | | | 67% | | | 11% | | | 67% | | | 12% | | | | 10% | | | | 6% | | | |  |
| **HR** | 1.40 [0.64;3.07] | | | 0.80 [0.52;1.24] | | | 1.40 [0.64;3.06] | | | 1.03 [0.68;1.55] | | | 0.76 [0.45;1.28] | | | 1.05 [0.69;1.58] | | | 0.85 [0.53;1.35] | | | | 0.72 [0.44;1.18] | | | | 0.58 [0.30;1.10] | | | |  |
| ^§^The three eligibility criteria are 1) age at baseline between 18 and 55 year, 2) an EDSS score at baseline between 0 and 5, and 3) having had at least one relapse in the year prior to baseline. ^†^This is an adjusted eligibility criteria where a patient had no relapses in the year prior to baseline as opposed to CONFIRM’s criteria of having at least one relapse in the year prior to baseline. | | | | | | | | | | | | | | | | | | | | | | | | | | | | | | |  |

## Table S15. Baseline characteristics for the pre/post 2012 GA population

A) Subgroup of GA patients that initiated treatment prior to 2012

| ITT | *Strict trial-emulation* | | | *Pragmatic scenario* | | |
| --- | --- | --- | --- | --- | --- | --- |
| **Eligibility criteria** | **Age** | **EDSS** | **ARR** | **Age** | **EDSS** | **ARR** |
|  | 18-55 | ≤5 | ≥1 |  |  |  |
|  | **DMF** | **GA** | **p** | **DMF** | **GA** | **p** |
| **n** | 111 | 147 |  | 1072 | 523 |  |
| **Person-years** | 204 | 271 |  | 1974 | 963 |  |
| **age (mean (SD))** | 34.4 (8.2) | 35.8 (8.5) | 0.19 | 38.1 (10.7) | 40.4 (10.6) | **<0.001** |
| **Sex (%)*** |  |  | 0.53 |  |  | 0.01 |
| **Female (%)** | 81 (73) | 114 (78) |  | 754 (70) | 400 (76) |  |
| **Male (%)** | 30 (27) | 34 (23) |  | 318 (30) | 123 (24) |  |
| **EDSS at baseline  (mean (SD))** | 1.7 (1.1) | 1.9 (1.3) | 0.23 | 1.6 (1.3) | 2.0 (1.5) | **<0.001** |
| **Number of relapses in previous 12 months (mean (SD))** | 1.3 (0.5) | 1.5 (0.9) | **0.005** | 0.1 (0.4) | 0.5 (0.8) | **<0.001** |
| **Number of relapses in previous 12 months (%)*** |  |  | **0.01** |  |  | **<0.001** |
| **0** | 84 (76) | 101 (68) |  | 955 (89) | 363 (69) |  |
| **1** | 24 (22) | 25 (17) |  | 93 (9) | 108 (21) |  |
| **2** | 3 (3) | 15 (10) |  | 21 (2) | 30 (6) |  |
| **3** | 0 (0) | 7 (5) |  | 2 (0) | 15 (3) |  |
| **4** | 0 (0) | 0 (0) |  | 0 (0) | 7 (1) |  |
| **Number of pre-baseline DMT treatments (%)*** |  |  | **0.003** |  |  | **<0.001** |
| **0** | 87 (78) | 86 (59) |  | 745 (70) | 287 (55) |  |
| **1** | 19 (17) | 50 (34) |  | 227 (21) | 204 (40) |  |
| **2** | 4 (4) | 12 (8) |  | 77 (7) | 31 (6) |  |
| **3** | 1 (1) | 0 (0) |  | 18 (2) | 1 (0) |  |
| **4** | 0 (0) | 0 (0) |  | 5 (1) | 0 (0) |  |
| **Of which firstline therapies (%)*** |  |  | **0.003** |  |  | **<0.001** |
| **0** | 89 (80) | 86 (59)) |  | 756 (70) | 288 (55) |  |
| **1** | 18 (16) | 51 (35) |  | 255 (24) | 209 (40) |  |
| **2** | 4 (4) | 11 (7) |  | 55 (5) | 25 (5) |  |
| **3** | 0 (0) | 0 (0) |  | 6 (1) | 1 (0) |  |
| **Of which secondline therapies (%)*** |  |  | 0.36 |  |  | **<0.001** |
| **0** | 108 (7) | 146 (99) |  | 1017 (95) | 516 (99) |  |
| **1** | 2 (2) | 1 (1) |  | 38 (4) | 7 (1) |  |
| **2** | 1 (1) | 0 (0) |  | 16 (2) | 0 (0) |  |
| **Years since diagnosis (SD)** | 1.6 (3.8) | 2.4 (3.9) | 0.07 | 2.9 (5.0) | 4.1 (5.8) | **<0.001** |
| *Due to rounding of the pooled estimates after imputation, the percentages may not add up to exactly 100%, and the summation of categorical variables may not be equal to *n*. | | | | | | |

B) Subgroup of GA patients that initiated treatment after 2012

| ITT | *Strict trial-emulation* | | | *Pragmatic scenario* | | |
| --- | --- | --- | --- | --- | --- | --- |
| **Eligibility criteria** | **Age** | **EDSS** | **ARR** | **Age** | **EDSS** | **ARR** |
|  | 18-55 | ≤5 | ≥1 |  |  |  |
|  | **DMF** | **GA** | **p** | **DMF** | **GA** | **p** |
| **n** | 111 | 30 |  | 1072 | 236 |  |
| **Person-years** | 204 | 55 |  | 1974 | 435 |  |
| **age (mean (SD))** | 34.4 (8.2) | 37.3 (7.4) | 0.07 | 38.1 (10.7) | 38.2 (11.0) | 0.88 |
| **Sex (%)*** |  |  | **<0.001** |  |  | **0.02** |
| **Female (%)** | 81 (73) | 23 (76) |  | 754 (70) | 185 (78) |  |
| **Male (%)** | 30 (27) | 7 (23) |  | 318 (30) | 51 (22) |  |
| **EDSS at baseline  (mean (SD))** | 1.7 (1.1) | 1.6 (1.2) | 0.59 | 1.6 (1.3) | 1.5 (1.3) | 0.88 |
| **Number of relapses in previous 12 months (mean (SD))** | 1.3 (0.5) | 1.2 (0.5) | 0.70 | 0.1 (0.4) | 0.2 (0.5) | 0.41 |
| **Number of relapses in previous 12 months (%)*** |  |  | 0.84 |  |  | 0.21 |
| **0** | 84 (76) | 24 (80) |  | 955 (89) | 205 (87) |  |
| **1** | 24 (22) | 5 (17) |  | 93 (9) | 24 (10) |  |
| **2** | 3 (3) | 1 (3) |  | 21 (2) | 6 (3) |  |
| **3** | 0 (0) | 0 (0) |  | 2 (0) | 1 (0) |  |
| **Number of pre-baseline DMT treatments (%)*** |  |  | 0.96 |  |  | 0.46 |
| **0** | 87 (78) | 24 (80) |  | 745 (70) | 165 (70) |  |
| **1** | 19 (17) | 5 (17) |  | 227 (21) | 55 (23) |  |
| **2** | 4 (4) | 1 (3) |  | 77 (7) | 15 (6) |  |
| **3** | 1 (1) | 0 (0) |  | 18 (2) | 1 (0) |  |
| **4** | 0 (0) |  |  | 5 (1) | 0 (0) |  |
| **Of which firstline therapies (%)*** |  |  | 0.57 |  |  | 0.38 |
| **0** | 89 (80) | 25 (83) |  | 756 (70) | 170 (72) |  |
| **1** | 18 (16) | 5 (17) |  | 255 (24) | 59 (25) |  |
| **2** | 4 (4) | 0 (0) |  | 55 (5) | 6 (3) |  |
| **3** | 0 (0) | 0 (0) |  | 6 (1) | 1 (0) |  |
| **Of which secondline therapies (%)*** |  |  | 0.31 |  |  | 0.21 |
| **0** | 108 (7) | 28 (93) |  | 1017 (95) | 223 (94) |  |
| **1** | 2 (2) | 2 (7) |  | 38 (4) | 12 (5) |  |
| **2** | 1 (1) | 0 (0) |  | 16 (2) | 1 (0) |  |
| **Years since diagnosis (SD)** | 1.6 (3.8) | 1.7 (3.6) | 0.85 | 2.9 (5.0) | 2.2 (4.0) | 0.05 |
| *Due to rounding of the pooled estimates after imputation, the percentages may not add up to exactly 100%, and the summation of categorical variables may not be equal to *n*. | | | | | | |

## Table S16. Outcome table – subgroup analysis pre and post 2012 GA subgroup

A) Subgroup with GA patients initiating treatment prior to 2012

| **ITT** | **Strict scenario** | | | **Pragmatic scenario** | | | **EDSS & ARR restricted** | | | **Age & ARR restricted** | | | **Age & EDSS restricted** | | | **pre-index ARRs restricted** | | | **Age restricted** | | | | **EDSS restricted** | | | | **pre-index ARR=0** | | | |  |
| --- | --- | --- | --- | --- | --- | --- | --- | --- | --- | --- | --- | --- | --- | --- | --- | --- | --- | --- | --- | --- | --- | --- | --- | --- | --- | --- | --- | --- | --- | --- | --- |
| **Eligibility criteria**^§^ | **Age** | **EDSS** | **ARR** | **Age** | **EDSS** | **ARR** | **Age** | **EDSS** | **ARR** | **Age** | **EDSS** | **ARR** | **Age** | **EDSS** | **ARR** | **Age** | **EDSS** | **ARR** | | **Age** | **EDSS** | **ARR** | | **Age** | **EDSS** | **ARR** | | **Age** | **EDSS** | **ARR** | |
|  | **18-55** | **≤5** | **≥1** |  |  |  |  | **≤5** | **≥1** | **18-55** |  | **≥1** | **18-55** | **≤5** |  |  |  | **≥1** | | **18-55** |  |  | |  | **≤5** |  | |  |  | **0^†^** | |
| **n** | 258 | | | 1595 | | | 269 | | | 264 | | | 1437 | | | 275 | | | 1460 | | | | 1561 | | | | 1320 | | | |  |
| **person-years** | 475 | | | 2937 | | | 495 | | | 486 | | | 2646 | | | 506 | | | 2688 | | | | 2874 | | | | 2430 | | | |  |
| **ARR GA** | 1.22 [0.32;4.56] | | | 0.36 [0.12;1.11] | | | 1.24 [0.36; 4.20] | | | 1.21 [0.32;4.50] | | | 0.31 [0.08;1.11] | | | 1.23 [0.36;4.15] | | | 0.31 [0.09;1.09] | | | | 0.35 [0.11;1.09] | | | | 0.20 [0.03;1.51] | | | |  |
| **ARR DMF** | 0.91 [0.25;3.27] | | | 0.21 [0.08;0.60] | | | 0.94 [0.29;3.03] | | | 0.94 [0.26;3.34] | | | 0.18 [0.05;0.60] | | | 0.97 [0.30;3.10] | | | 0.19 [0.06;0.61] | | | | 0.20 [0.07;0.57] | | | | 0.10 [0.02;0.64] | | | |  |
| **ARRR** | 0.75 [0.43;1.30] | | | 0.59 [0.35;1.00] | | | 0.76 [0.44;1.32] | | | 0.78 [0.45;1.34] | | | 0.58 [0.34;1.01] | | | 0.79 [0.46;1.36] | | | 0.61 [0.36;1.05] | | | | 0.57 [0.33;0.97] | | | | 0.51 [0.19;1.35] | | | |  |
| **Proportion relapsed after 2 years (GA)** | 65% | | | 20% | | | 64% | | | 64% | | | 20% | | | 64% | | | 20% | | | | 20% | | | | 14% | | | |  |
| **Proportion relapsed after 2 years (DMF)** | 68% | | | 12% | | | 67% | | | 68% | | | 13% | | | 67% | | | 13% | | | | 12% | | | | 5% | | | |  |
| **HR** | 1.09 [0.85;1.40] | | | 0.52 [0.43;0.64] | | | 1.10 [0.86;1.40] | | | 1.10 [0.86;1.41] | | | 0.55 [0.45;0.68] | | | 1.10 [0.86;1.41] | | | 0.56 [0.45;0.68] | | | | 0.52 [0.42;0.63] | | | | 0.31 [0.23;0.42] | | | |  |
| ^§^The three eligibility criteria are 1) age at baseline between 18 and 55 year, 2) an EDSS score at baseline between 0 and 5, and 3) having had at least one relapse in the year prior to baseline. ^†^This is an adjusted eligibility criteria where a patient had no relapses in the year prior to baseline as opposed to CONFIRM’s criteria of having at least one relapse in the year prior to baseline. | | | | | | | | | | | | | | | | | | | | | | | | | | | | | | |  |

B) Subgroup with GA patients initiating treatment from 2012 onwards

| **ITT** | **Strict scenario** | | | **Pragmatic scenario** | | | **EDSS & ARR restricted** | | | **Age & ARR restricted** | | | **Age & EDSS restricted** | | | **pre-index ARRs restricted** | | | **Age restricted** | | | | **EDSS restricted** | | | | **pre-index ARR=0** | | | |  |
| --- | --- | --- | --- | --- | --- | --- | --- | --- | --- | --- | --- | --- | --- | --- | --- | --- | --- | --- | --- | --- | --- | --- | --- | --- | --- | --- | --- | --- | --- | --- | --- |
| **Eligibility criteria**^§^ | **Age** | **EDSS** | **ARR** | **Age** | **EDSS** | **ARR** | **Age** | **EDSS** | **ARR** | **Age** | **EDSS** | **ARR** | **Age** | **EDSS** | **ARR** | **Age** | **EDSS** | **ARR** | | **Age** | **EDSS** | **ARR** | | **Age** | **EDSS** | **ARR** | | **Age** | **EDSS** | **ARR** | |
|  | **18-55** | **≤5** | **≥1** |  |  |  |  | **≤5** | **≥1** | **18-55** |  | **≥1** | **18-55** | **≤5** |  |  |  | **≥1** | | **18-55** |  |  | |  | **≤5** |  | |  |  | **0^†^** | |
| **n** | 141 | | | 1308 | | | 144 | | | 143 | | | 1195 | | | 146 | | | 1209 | | | | 1287 | | | | 1162 | | | |  |
| **person-years** | 260 | | | 2408 | | | 265 | | | 263 | | | 2200 | | | 269 | | | 2226 | | | | 2369 | | | | 2139 | | | |  |
| **ARR GA** | 0.85 [0.06;12.09] | | | 0.28 [0.06;1.25] | | | 0.76 [0.08;7.54] | | | 0.87 [0.06;12.16] | | | 0.18 [0.03;1.07] | | | 0.78 [0.08;7.58] | | | 0.18 [0.03;1.06] | | | | 0.28 [0.06;1.24] | | | | 0.19 [0.01;2.49] | | | |  |
| **ARR DMF** | 0.66 [0.06;7.71] | | | 0.17 [0.04;0.65] | | | 0.60 [0.08;4.70] | | | 0.68 [0.06;7.78] | | | 0.11 [0.02;0.57] | | | 0.62 [0.08;4.75] | | | 0.11 [0.02;0.58] | | | | 0.16 [0.04;0.64] | | | | 0.09 [0.01;0.98] | | | |  |
| **ARRR** | 0.78 [0.27;2.22] | | | 0.59 [0.26;1.35] | | | 0.78 [0.28;2.22] | | | 0.78 [0.27;2.23] | | | 0.61 [0.26;1.40] | | | 0.80 [0.29;2.24] | | | 0.62 [0.27;1.45] | | | | 0.58 [0.25;1.30] | | | | 0.47 [0.11;2.05] | | | |  |
| **Proportion relapsed after 2 years (GA)** | 70% | | | 16% | | | 70% | | | 70% | | | 16% | | | 71% | | | 16% | | | | 16% | | | | 9% | | | |  |
| **Proportion relapsed after 2 years (DMF)** | 67% | | | 11% | | | 68% | | | 68% | | | 12% | | | 69% | | | 12% | | | | 11% | | | | 6% | | | |  |
| **HR** | 0.94 [0.59;1.51] | | | 0.67 [0.48;0.93] | | | 0.94 [0.59;1.51] | | | 0.94 [0.59;1.52] | | | .068 [0.47;0.98] | | | 0.95 [0.59;1.51] | | | 0.70 [0.49;1.00] | | | | 0.65 [0.47;0.91] | | | | 0.64 [0.42;1.00] | | | |  |
| ^§^The three eligibility criteria are 1) age at baseline between 18 and 55 year, 2) an EDSS score at baseline between 0 and 5, and 3) having had at least one relapse in the year prior to baseline. ^†^This is an adjusted eligibility criteria where a patient had no relapses in the year prior to baseline as opposed to CONFIRM’s criteria of having at least one relapse in the year prior to baseline. | | | | | | | | | | | | | | | | | | | | | | | | | | | | | | |  |

# Appendices

## Appendix 1. Rationale for emulating the CONFIRM target trial

To test the two-step target trial emulation approach, CONFIRM was selected as the target trial to emulate following both strict trial-like and pragmatic eligibility criteria. CONFIRM was the result from various feasibility tests to come to a suitable randomized active controlled clinical trial in the setting of relapsing-remitting-multiple sclerosis:

1.	Relapsing remitting multiple sclerosis was chosen as disease setting, since most (if not all) of the disease modifying therapies follow a static treatment regime. This was selected as a hard requirement over (dynamic treatment regimens) to not further complicate the methodological interpretation of the estimates resulting from our two-step emulation. Moreover, there are relatively few literature-confirmed confounders, of which was reasonably assumed that none of them were time-varying over a period of 96 weeks. Furthermore, the endpoints, such as relapses, are relatively straightforward to observe; and lastly, due to the incidence and prevalence of relapsing remitting multiple sclerosis in Sweden, a sufficient sample size was feasible.

2.	CONFIRM was thereafter selected as our target trial for various reasons:

a.	One of the requirements for the target trial was that it should have an active comparator arm and both the treatment and comparator should be firstline treatments, as to not further complicate the methodological interpretation of the estimates resulting from the two-step emulation.

b.	CONFIRM was a relatively big and, above all, recent pivotal trial, while still having had enough time passed since marketing authorization of the study drugs to ensure enough prescriptions and events in the observational data until end of data collection in 2024.

c.	CONFIRM was deemed as relatively low risk-of-bias in our previous systematic review and network meta-analysis (DOI: 10.1002/pds.5810), as compared to the other trials that passed criteria 2a and 2b above.

The subgroups were deliberately chosen because they are commonly ignored in pivotal RCTs (elderly and those with severe disease), and because relapse-free patients make-up a large fraction of the patients with relapsing-remitting multiple sclerosis from 2010 onwards.

## Appendix 2. TARGET checklist

| Item no. | | | | Checklist item | | | | | Location reported | |
| --- | --- | --- | --- | --- | --- | --- | --- | --- | --- | --- |
| Abstract | | | | | | | | | | |
| 1 | a | | | | Identify that the study attempts to emulate a target trial using observational data. State the study objectives and briefly summarize the specified target trial. | | | | Abstract (background & methods) | |
|  | b | | | | Report the data sources used for emulation. | | | | Abstract (methods) | |
|  | c | | | | Summarize key assumptions, statistical methods, findings and conclusions. | | | | Abstract (methods, results, discussion) | |
| Introduction | | | | | | | | | | |
| 2 | Background | | | | Describe the scientific background of the study and the gap in knowledge. | | | | Introduction (first three paragraphs) | |
| 3 | Causal question | | | | Summarize the causal question. | | | | Introduction (last paragraph) | |
| 4 | Rationale | | | | Describe the rationale for emulating a target trial with the available data. Cite randomized trials informing the design of the target trial if applicable. | | | | Introduction (last paragraph) | |
| **Methods** | | | | | | | | | | |
| 5 | Data sources | | Cite the data sources contributing to the analyses and for each one describe the following: original purpose, type, the geographic locations, setting and time-period. If relevant, describe how the data were linked or pooled. | | | | | | Methods (Data Source) | |
| 6 | Target trial specification  Specify the components of the target trial protocol that would answer the causal question. | | | | | 7 | **Target trial emulation**  Describe how the components of the target trial protocol were emulated with the observational data, including how all variables were measured or ascertained. | | **Location item 6** Methods (Data source); Table 1 | **Location item 7** Methods (second paragraph onwards); Table 1 |
|  | Eligibility criteria | | | | |  | Eligibility criteria | | Methods (Data source)Table 1 | Methods (Population); Table 1 |
|  | a | Describe the eligibility criteria. | | | |  | a | Describe how the eligibility criteria were operationalized with the data. |  |  |
|  | **Treatment strategies** | | | | |  | **Treatment strategies** | | Methods (Data source); Table 1 | Methods (Treatment & follow-up); Table 1 |
|  | b | Describe the treatment strategies that would be compared. | | | |  | b | Describe how the treatment strategies were operationalized with the data. |  |  |
|  | **Assignment procedures** | | | | |  | **Assignment procedures** | | Methods (Data source); Table 1 | Methods (Treatment & follow-up); Table 1 |
|  | c | Report that eligible individuals would be randomly assigned to treatment strategies and may be aware of their treatment allocation. | | | |  | c | Describe how assignment to treatment strategies was operationalized with the data. |  |  |
|  | **Follow-up** | | | | |  | **Follow-up** | | Methods (Data source); Table 1 | Methods (Treatment & follow-up); Table 1 |
|  | d | Clarify that follow-up would start at time of assignment to the treatment strategies. Specify when follow-up would end. | | | |  | d | Clarify that follow-up starts at the time individuals were assigned to the treatment strategies. Describe how the end of follow-up was operationalized with the data. |  |  |
|  | **Outcomes** | | | | |  | **Outcomes** | | Methods (Data source); Table 1 | Methods (Outcome); Table 1 |
|  | e | Describe the outcomes. | | | |  | e | Describe how the outcomes were operationalized with the data. |  |  |
|  | Causal contrasts | | | | |  | Causal contrasts | | Methods (Data source); Table 1 | Methods (Treatment & follow-up); Table 1 |
|  | f | Describe the causal contrasts of interest, including effect measures. | | | |  | f | Describe how the causal contrasts were operationalized with the data, including effect measures. |  |  |
|  | **Identifying assumptions** | | | | |  | **Identifying assumptions** | | Methods (Data Source) | Methods (Treatment & follow-up) |
|  | g | Describe assumptions that would be made to identify each causal estimand. Describe the variables, if any, related to these assumptions. | | | |  | g.i | For each causal estimand, describe assumptions made to identify it, including assumptions regarding baseline confounding due to lack of randomization. |  |  |
|  |  |  | | | |  | g.ii | Describe how the variables related to these assumptions were operationalized with the data |  | Study protocol EUPAS1000000135 |
|  | **Data analysis plan** | | | | |  | **Data analysis plan** | | Table 1 | Methods (Statistical analysis); Table 1 |
|  | h | For each causal estimand, describe the data analysis procedures and any associated statistical modelling assumptions, including approaches for handling missing data. | | | |  | h.i | For each causal estimand, describe the data analysis procedures and any associated statistical modelling assumptions, including approaches for handling missing data. |  |  |
|  |  |  | | | |  | h.ii | For each causal estimand, describe any additional analyses conducted to assess the sensitivity of the results to the choice of operationalizations, assumptions and analysis. |  | Methods (Statistical analysis); Table 1 |
| **Results** | | | | | | | | | | |
| 8 | Participant selection | | | | Report numbers of individuals assessed for eligibility, eligible, and assigned to each treatment strategy. A flow diagram is strongly recommended. | | | | Results (Patients); Figure 1 | |
| 9 | Baseline data | | | | Describe the distribution of characteristics of individuals at baseline, by treatment strategy. | | | | Results (Patients); Table 2 | |
| 10 | Follow-up | | | | Summarize length of follow-up and describe reasons for end of follow-up for each treatment strategy and causal contrast. | | | | Table 3; Supplementary Information Tabls S4-S6. | |
| 11 | Missing data | | | | Describe the frequency of missing data in all variables, by treatment strategy when applicable. | | | | Results (Patients), missingness per treatment strategy not applicable due to imputation being done before applying the eligibility criteria. | |
| 12 | Outcomes | | | | Describe the frequency or distribution of each outcome, by treatment strategy. | | | | Table 3 | |
| 13 | Effect estimates | | | | Report the effect estimates for each causal contrast with corresponding measures of precision, including both absolute and relative measures of effect, when applicable. | | | | Results (Annualized relapse rate; Probability of experiencing a first relapse); Table 3 | |
| 14 | Additional analyses | | | | Report results of all analyses to assess the sensitivity of the estimates to choices in operationalizations, assumptions and analysis. | | | | Results (Sensitivity analysis) | |
| **Discussion** | | | | | | | | | | |
| 15 | Interpretation | | Provide an interpretation of the key findings. | | | | | | Discussion (first three paragraphs) | |
| 16 | Limitations | | Discuss the limitations of the study considering differences between the target trial and its emulation and the plausibility of assumptions, including assumptions regarding baseline confounding due to lack of randomization. | | | | | | Discussion, fifth to seventh paragraphs) | |
| **Other information** | | | | | | | | | | |
| **17** | Ethics | | | | Provide the institutional research board or ethics committee that approved the study and approval numbers, if relevant. | | | | Methods (Data source) | |
| **18** | Registration | | | | State whether, when and where the study protocol was registered. | | | | Methods (Data source); Study protocol EUPAS1000000135 | |
| **19** | Sharing of study materials | | | | Provide information on whether data, analytic code and/or other materials are accessible, and where and how they can be accessed. | | | | ✔ | |
| **20** | Funding sources | | | | Provide the sources of funding and detail the role of the funders in the design, conduct and reporting of the study. | | | | ✔ | |
| **21** | Conflicts of interest | | | | State any conflicts of interest and financial disclosures for all authors. | | | | ✔ | |

## Appendix 3. Rationale for not including MRI

MRI information was not considered in this study. The eligibility criterion in the CONFIRM RCT to include patients with a gadolinium-enhancing T1-weighted lesion, detected in the six weeks before baseline (as alternative for the criterion of having had at least one relapse in the year prior to randomization), was not considered, since it was expected that very few patients would have had an eligible detected gadolinium-enhancing lesion within this short time-window registered in the observational data. Therefore, our study population in the strict trial-like scenario may differ from the original trial population, by overlooking patients without a relapse in the year prior to baseline, but with an active inflammation. These patients are, however, included in our pragmatic scenario.

MRI was neither considered in any other context, due to the heterogeneity of the data. In trial settings, lesions are often used as exploratory (or surrogate) endpoint for relapses. However, in these trials, lesions are often measured using a single, detailed, and predefined MRI protocol, whereas in multi-center observational setting, MRI data are more heterogeneous, and less clearly recorded: either being T1-weighted (enhanced with or without gadolinium) or T2-weighted, brain or spinal cord, differences in scanner-settings, and center-specific MRI protocols and updates of these MRI protocols over the multiple years this study covered. For T2-weighted MRI, follow-up times between consecutive MRI scans differ as well, resulting in discrepancies in newly/enlarged lesion detection. As a result, due to this heterogeneous MRI evidence, it is more complex to establish internal and external validity of the relapses through MRI-measurements in observational settings, especially as compared to trials.
